# Hdac8 Inhibitor Alleviates Transverse Aortic Constriction-Induced Heart Failure in Mice by Downregulating Ace1

**DOI:** 10.1155/2022/6227330

**Published:** 2022-01-27

**Authors:** Tingwei Zhao, Hae Jin Kee, Seung-Jung Kee, Myung Ho Jeong

**Affiliations:** ^1^Heart Research Center of Chonnam National University Hospital, Gwangju 61469, Republic of Korea; ^2^Hypertension Heart Failure Research Center, Chonnam National University Hospital, Gwangju 61469, Republic of Korea; ^3^Department of Laboratory Medicine, Chonnam National University, Medical School and Hospital, Gwangju 61469, Republic of Korea; ^4^Department of Cardiology, Chonnam National University Medical School, Gwangju 61469, Republic of Korea

## Abstract

**Background:**

Heart failure is characterized by activation of the renin-angiotensin-aldosterone system, which is involved in the regulation of cardiac hypertrophy and hypertension. Recently, we reported that Hdac8 inhibition alleviates isoproterenol-induced and angiotensin II-induced cardiac hypertrophy or hypertension in mice. Here, the effect and regulatory mechanisms of the Hdac8 selective inhibitor PCI34051 on pressure overload-induced heart failure were examined.

**Methods and Results:**

At week 6 posttransverse aortic constriction (TAC), mice were administered with PCI34051 (3, 10, or 30 mg/kg bodyweight/day) for 2 weeks. The therapeutic effects of PCI34051 on TAC-induced cardiac and lung hypertrophy were determined by examining the heart weight-to-bodyweight and lung weight-to-bodyweight ratios and the cross-sectional cardiomyocyte area. Echocardiography analysis revealed that PCI34051 mitigated TAC-induced decreased ejection fraction and fractional shortening. Additionally, the expression of Hdac8 was upregulated in the cardiac and pulmonary tissues of TAC mice. The expression levels of Ace1 and Agtr1 were upregulated, whereas those of Ace2 and Agtr2 were downregulated in TAC mice. PCI34051 treatment or *Hdac8* knockdown alleviated inflammation as evidenced by Rela downregulation and Nfkbia upregulation in mice, as well as in cardiomyocytes, but not in cardiac fibroblasts. Hdac8 overexpression-induced Rela pathway activation was downregulated in *Ace1* knockdown cells. Picrosirius red staining, real-time polymerase chain reaction, and western blotting analyses revealed that PCI34051 alleviated fibrosis and downregulated fibrosis-related genes. Moreover, PCI34051 or *Hdac8* knockdown in rat cardiac fibroblasts alleviated cardiac fibrosis through the Tgfb1-Smad2/3 pathway. The results of overexpression and knockdown experiments revealed that Hdac8 and Ace1 promote inflammation and fibrosis.

**Conclusions:**

Treatment with PCI34051 enhanced cardiac and lung functions in the TAC-induced heart failure mouse model. These data suggest that HDAC8 is a potential novel therapeutic target for heart failure accompanied by pathological lung diseases.

## 1. Introduction

The prevalence, mortality, and morbidity rates of heart failure, a multifactorial disease accompanied by structural and functional damages to the tissues [1, 2], are increasing annually worldwide, including the United States of America (USA) [3–5]. Heart failure is often accompanied by fibrosis, which is the tissue response to inflammation. Previous studies have reported that the levels of inflammatory cytokines, such as tumor necrosis factor-alpha (TNF*α*), interleukin-1 beta (IL-1*β*), and interleukin-6 (IL-6) are upregulated in patients with heart failure [6, 7] and in the transverse aortic constriction- (TAC-) induced heart failure mouse model [8]. Heart failure promotes the neurohormonal activation of the sympathetic nervous system and the renin-angiotensin-aldosterone system (RAAS), which plays a critical role in the regulation of cardiovascular processes [9, 10]. Angiotensin II, a key molecule in the RAAS, regulates cardiac contractility and promotes adverse remodeling in heart failure [11]. ACE1 catalyzes the conversion of angiotensin I into angiotensin II, while ACE2 catalyzes the conversion of angiotensin II into angiotensin (1–7) or angiotensin I to angiotensin (1–9), leading to cardioprotective effects on heart failure [12, 13]. ACE2 is also expressed in the lung vascular endothelial cells and consequently protects against acute lung failure [14, 15]. In mice, Ace2 deficiency leads to early cardiac hypertrophy [16], whereas Ace1 overexpression promotes atrial enlargement [17]. Therefore, the neurohormonal blockade using ACE inhibitors, angiotensin receptor blockers, and beta-blockers mitigates heart failure-induced cardiac remodeling [18, 19].

Recent studies have reported that histone deacetylases (HDACs) regulate gene expression. HDACs are classified into four classes. Class I HDACs comprise HDAC1, HDAC2, HDAC3, and HDAC8. Among class I HDACs, HDAC2 has been best characterized for its role in the modulation of cardiac hypertrophy [20, 21]. For example, the enzymatic activity but not the expression of HDAC2 promotes cardiac hypertrophy [22]. HDAC inhibitors, including trichostatin A and SK-7041, hypertrophy and heart failure [23–25]. HDAC3 increases postnatal cardiac myocyte proliferation but is not involved in cardiac hypertrophy [26]. Recently, we had reported that PCI34051, an HDAC8 selective inhibitor, mitigates angiotensin II-induced hypertension and isoproterenol-induced cardiac hypertrophy [27, 28]. Previous studies have demonstrated that HDAC8 regulates cardiac hypertrophy through the AKT/GSK3*β* pathway [29]. However, the role of HDAC8 in heart failure has not been elucidated. In contrast to class I HDACs, class II HDACs, such as HDAC5 and HDAC9, suppress cardiac hypertrophy [30].

This study demonstrated that pharmacological inhibition or downregulation of Hdac8 mitigates heart failure-related pathologies, including cardiac hypertrophy, pulmonary congestion, fibrosis, and inflammation in vivo (TAC-induced heart failure mouse model) and in vitro. The results of in vitro studies demonstrated that PCI34051 treatment or *Hdac8* or *Ace1* knockdown modulated inflammatory and fibrotic responses to transforming growth factor-beta 1 (TGF-*β*1) or TNF*α* stimulation. This is the first study to demonstrate the role of HDAC8 in the pathogenesis of heart failure.

## 2. Materials and Methods

### 2.1. Reagents

PCI34051 (10444) was purchased from Cayman Chemical Company (Ann Arbor, MI, USA). Anti-Actb (sc-47778), anti-Nppb (sc-271185), anti-Acta2 (sc-130617), anti-Ace1 (sc-20791), anti-Ace2 (sc-390851), and anti-Tgfb1 (sc-146) antibodies were obtained from Santa Cruz Biotechnology (Dallas, TX, USA). Anti-phospho-Smad2/3 (8828), anti-Smad2/3 (3102), anti-Rela (8242), and anti-Nfkbia (4812) antibodies were purchased from Cell Signaling Technology (Danvers, MA, USA). Anti-Nppa and anti-Hdac8 (ab187139) antibodies were purchased from GeneTex (GTX109255; Irvine, CA, USA) and Abcam (Cambridge, UK), respectively. Anti-Fn1 (MA5-11981) and anti-Ccl2 (PA5-34505) antibodies were purchased from Thermo Fisher Scientific Inc. (Waltham, MA, USA).

### 2.2. Establishment of the TAC-Induced Heart Failure Mouse Model

All animal experiments were approved by the Animal Experimental Committee of Chonnam National University Medical School (CNUH IACUC-18023) and conducted according to the Guide for the Care and Use of Laboratory Animals (US National Institutes of Health Publications, 8^th^ edition, 2011). Male ICR mice (Orient, South Korea) aged 6 weeks were maintained in a 12 h light/dark cycle under specific pathogen-free conditions. The mouse skin was disinfected with ethyl alcohol, and the mice were anesthetized by intraperitoneally injecting a mixture of ketamine (120 mg/kg bodyweight) and xylazine (6.2 mg/kg body weight). The anesthetized mice were fixed on the transparent board in a supine position and connected to a small animal ventilator through laryngotracheal intubation. The lung characteristics of the mice were as follows: tidal volume, 0.1–0.3 mL and respiratory rate, 125–150 breaths/min. The aortic arch was exposed, and the thymus was removed. The transverse aortic arch was tied with a 7-0 silk suture between the brachiocephalic and left common carotid arteries using an overlaying 27G needle. The constriction needle was carefully removed, the chest and skin incision wounds were closed using a 4-0 silk suture, and the ventilator was disconnected. Mice were placed on warm pads until they woke up after surgery. The sham group underwent the same surgical procedure without aortic banding. The animals were randomized into the following six groups (5–6 mice per group): sham+vehicle, sham+PCI34051 (10 mg/kg bodyweight/day), TAC+vehicle, TAC+PCI34051 (3 mg/kg bodyweight/day), TAC+PCI34051 (10 mg/kg bodyweight/day), and TAC+PCI34051 (30 mg/kg bodyweight/day). PCI34051 was intraperitoneally administered daily for 2 weeks. All animals were sacrificed using CO_2_.

### 2.3. Echocardiography

To evaluate the left ventricular functions, echocardiography was performed using a Vivid S5 echocardiography system (GE Healthcare, Chicago, IL, USA) equipped with a 13 MHz linear array transducer as described previously [28]. Before cardiac function measurements, mice were anesthetized by intraperitoneally administering tribromoethanol (Avertin; 114 mg/kg bodyweight). M-mode (2-D guided) images and parameters were acquired from the long-axis view of the left ventricle at the level of the papillary muscles.

### 2.4. Histological Analysis and Picrosirius Red Staining

Mouse cardiac tissues were fixed with 3.7% paraformaldehyde and embedded in paraffin. The paraffin-embedded tissues were cut into 4 *μ*m thick sections. The sections were deparaffinized with xylene, rehydrated in a graded alcohol series, and subjected to hematoxylin and eosin (H&E) staining to measure the size of cardiomyocytes in the myocardial tissue [31]. Quantification of cell size was performed using the NIS Elements software (Nikon Eclipse 80*i* microscope, Tokyo, Japan).

Picrosirius red (Abcam) staining was performed to evaluate cardiac fibrosis. The rehydrated cardiac tissues were stained with Picrosirius red solution for 1 h. The samples were quickly washed twice with 0.5% acetic acid solution and rinsed with absolute alcohol for 1 min. The sections were mounted with Canada balsam and imaged using a microscope (Nikon, Tokyo, Japan) at 400x magnification.

### 2.5. Isolation and Cell Culture of Primary Neonatal Cardiac Fibroblasts

Cardiac fibroblasts were isolated from rat neonatal hearts (15 pups) [32]. Briefly, the atrium was removed, finely chopped using scissors, and digested with 0.1% collagenase II in 1× ADS buffer (116 mM NaCl, 20 mM HEPES, 10 mM NaH_2_PO_4_, 5.5 mM glucose, 5 mM KCl, and 0.8 mM MgSO_4_) at 37°C and 120 rpm on a shaker for 2 h. The cells were neutralized using fetal bovine serum (FBS, final concentration 10%) and centrifuged at 1200 rpm for 3 min. Fibroblasts were cultured in Dulbecco's modified Eagle's medium supplemented with 10% FBS and 1× antibiotic-antimycotic solution at 37°C in an incubator. Passage 2 fibroblasts were used for the experiments. Cardiac fibroblasts were pretreated with TGF-*β*1 (10 ng/mL) for 1 h and cultured in the presence of vehicle (0.1% dimethyl sulfoxide (DMSO)) or PCI34051 (10 *μ*M) for 8 h. To investigate the roles of Hdac8 or Ace1 in fibrosis, fibroblasts were transfected with control short-interfering RNAs (si-RNAs) or siRNAs against *Hdac8* (si-Hdac8) or si-Ace1 and incubated with TGF-*β*1 (10 ng/mL) for 9 h.

### 2.6. Quantitative Real-Time Polymerase Chain Reaction (qRT-PCR)

Total RNAs were isolated from the cardiac tissues using TRIzol reagent (Invitrogen/Life Technologies, Carlsbad, CA, USA). The RNA concentration was determined by measuring the absorbances of the sample at wavelengths of 260 and 280 nm. The isolated RNA (1 *μ*g) was reverse-transcribed into complementary DNA using TOPscript RT DryMIX (Enzynomics, Daejeon, South Korea). qRT-PCR analysis was performed using the SYBR Green PCR kit and specific primers. The relative mRNA levels were determined using the 2^−∆∆Ct^ method. The sequence of primers used in qRT-PCR analysis is shown in Supplementary Table 1.

### 2.7. Western Blotting

Total proteins were isolated from the cardiac and pulmonary tissues using a radioimmunoprecipitation assay buffer as described previously [28]. The protein concentration was measured using the bicinchoninic protein assay kit. Equal amounts of proteins were subjected to sodium dodecyl sulfate-polyacrylamide gel electrophoresis. The resolved proteins were transferred to a polyvinylidene difluoride membrane (pore size: 0.45 *μ*m; Merk Millipore, MA, USA). The membrane was blocked with 5% skim milk in Tris-buffered saline containing Tween-20 (TBST) (20 mM Tris, 200 mM NaCl, and 0.04% Tween 20) for 1 h at 25°C and probed with the primary antibodies (1 : 1000) overnight at 4°C. Next, the membrane was washed thrice with TBST for 5 min and incubated with the anti-rabbit or anti-mouse horseradish peroxidase-conjugated secondary antibodies (1 : 3000) for 1 h at 25°C. Immunoreactive signals were detected using Immobilon western blotting detection reagents (EMD Millipore, Billerica, MA, USA). The intensities of the protein bands were quantified using ImageJ software (https://imagej.net/).

### 2.8. Transfection

To overexpress Hdac8, H9c2 cells were transfected with 1.6 *μ*g of *pCMV-HA-myc* or *pCMV-Hdac8-HA-myc* plasmids for 2 days using Lipofectamine and PLUS reagents, following the manufacturer's instructions. Additionally, the H9c2 cells were transfected with 1.6 *μ*g of *pCMV6-SPORT6* or *pCMV6-SPORT6-Ace1* plasmids for 2 days to overexpress Ace1. The *pCMV6-SPORT6-Ace1* clone was obtained from the Korea Human Gene Bank, Medical Genomics Research Center, KRIBB, Korea.

To knockdown *Hdac8* or *Ace1*, H9c2 cells were transfected with 100 nM control siRNAs (cat no. SN-1003, Bioneer, Daejeon, South Korea), si-Hdac8 (cat no. L-096589-02-0005, Dharmacon, Lafayette, CO, USA), or si-Ace1 (product name 24310, Bioneer) using RNAiMAX reagent.

The siRNA sequences were as follows: control sense, 5′-CCU ACG CCA AUU UCG U-3′ and control antisense, 5′-ACG AAA UUG GUG GCG UAG G-3′; si-Ace1 #1, 5′-CUC AGU AAU GAA GCC UAC A-3′; si-Ace1 #2, 5′-CAU UUG ACG UGA GCA ACU U-3′; si-Ace1 #3, 5′-ACA AAC CCA ACC UCG AUG U-3′; si-Hdac8 #1: 5′-UAG AAU AUG GAC UAG GUU A-3′; si-Hdac8 #2, 5′-GAU CCA AUG UGC UCC UUU A-3′; si-Hdac8 #3, 5′-CAG CAU AUG GUC CUG AUU A-3′; and si-Hdac8 #4: 5′-CAG AAG GGA UAU UUG ACU A-3′.

### 2.9. Nuclear and Cytoplasmic Protein Extraction

H9c2 cells pretreated with TNF*α* (50 ng/mL) for 1 h were incubated with vehicle (0.1% DMSO) or PCI34051 (10 *μ*M) for 5 h. The cytoplasmic extracts were prepared using the hypotonic lysis buffer (10 mM HEPES (pH 7.9), 10 mM KCl, 10 mM EDTA, 1 mM dithiothreitol (DTT), 0.4% IGEPAL, and 1× protease inhibitor). The extracts were incubated on ice for 10 min on a rocker and centrifuged at 13000 rpm and 4°C for 5 min. The supernatant was stored as cytoplasmic extracts. The nuclear pellets were resuspended in a high salt buffer (10 mM HEPES [pH 7.9], 400 mM NaCl, 1 mM EDTA, 1 mM DTT, and 1× protease inhibitor) and incubated on a rotating shaker on ice for 2 h. The samples were centrifuged at 13000 rpm and 4°C for 5 min to obtain the nuclear extracts.

### 2.10. Statistical Analysis

All data are represented as mean ± standard error. The means of two groups were compared using Student's *t*-test, whereas those of three or more groups were compared using one-way analysis of variance, followed by a Bonferroni multiple comparison test. Differences were considered significant at *P* < 0.05. All statistical analyses were performed using GraphPad Prism version 8.0.2 (GraphPad Software, La Jolla, CA, USA).

## 3. Results

### 3.1. PCI34051 Alleviates Cardiac Hypertrophy and Restores Cardiac Function in the TAC-Induced Heart Failure Mouse Model

**Figure 1:**
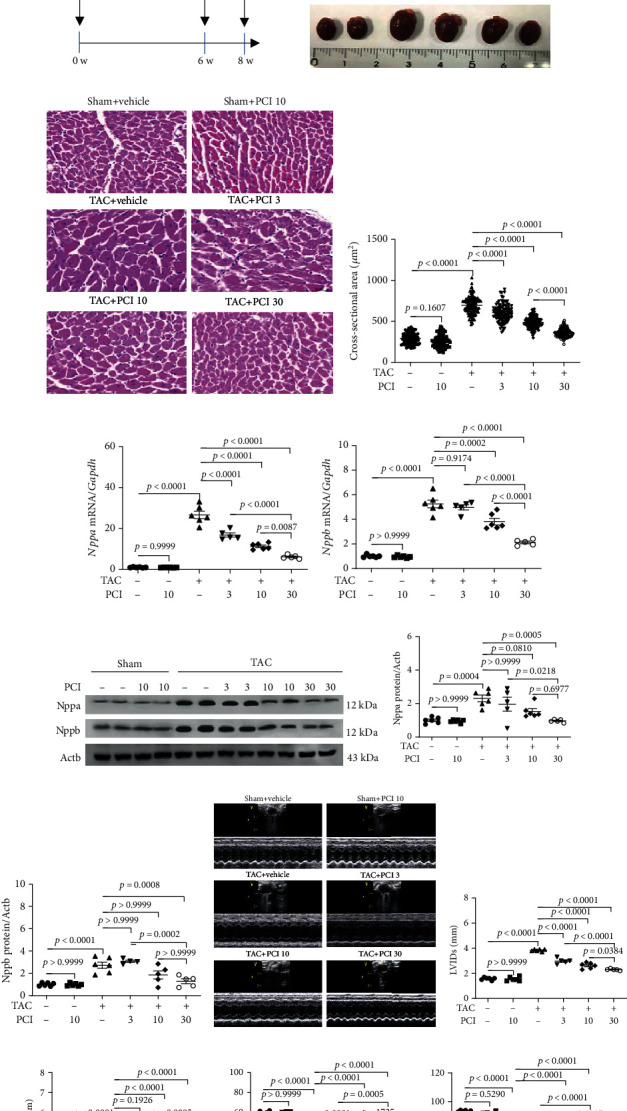
PCI34051 alleviates cardiac hypertrophy and left ventricular dysfunction in transverse aortic constriction- (TAC-) induced heart failure. (a) Schematic diagram of the TAC-induced heart failure mouse model and the PCI34051 treatment schedule. TAC promoted heart failure in mice. At week 6 post-TAC, mice were administered with vehicle or PCI34051 for 2 weeks as described in Materials and Methods. (b) Heart weight-to-bodyweight (HW/BW) ratio (*n* = 5–6 mice per group; A) and representative images (B) of heart from the sham+vehicle, sham+PCI34051 (10 mg/kg bodyweight/day), TAC+vehicle, and TAC+PCI34051 (3, 10, or 30 mg/kg bodyweight/day) groups. (c) Representative images of mouse cardiac tissues stained with hematoxylin and eosin (*n* = 5–6 mice per group). Scale bar = 50 *μ*m. (d) Quantification of cardiomyocyte cross-sectional area of samples described in (b). (e, f) mRNA levels of *Nppa* and *Nppb* were determined using quantitative real-time polymerase chain reaction and normalized to those of *Gapdh*. (g) Nppa and Nppb levels in the cardiac tissues of mice described in (b). Representative western blot images. (h, i) Quantification of Nppa and Nppb levels. (j–n) Representative M-mode echocardiograms and parameters in mice. Quantification of left ventricular internal dimension end-systolic (LVIDs) and left ventricular internal dimension end-diastolic (LVIDd), fractional shortening (FS), and ejection fraction (EF) (*n* = 5–6 mice per group).

Cardiac hypertrophy is often accompanied by hypertension and heart failure [33, 34]. We investigated the effects of different concentrations of PCI34051 (3, 10, and 30 mg/kg bodyweight/day), an HDAC8 selective inhibitor, on concomitant cardiac hypertrophy using the TAC-induced heart failure mouse model. The timeline of the experiments is shown in Figure 1(a). The heart size in the TAC group was markedly higher than that in the sham group. Treatment with PCI34051 at a dose of 10 mg/kg bodyweight/day did not affect the heart weight-to-bodyweight (HW/BW) ratio in the sham group. In contrast, all three doses of PCI34051 significantly mitigated the TAC-induced enhanced HW/BW ratio. The HW/BW ratio in the group treated with PCI34051 at a dose of 30 mg/kg bodyweight/day was lower than that in the groups treated with PCI34051 at doses of 3 and 10 mg/kg bodyweight/day (Figure 1(b)).

Next, the size of cardiomyocytes in the H&E-stained cardiac tissues was examined. The cross-sectional area was not significantly different between the vehicle-treated sham and PCI34051-treated sham groups. However, PCI34051 dose dependently decreased the cross-sectional area of cardiomyocytes in the TAC-treated groups (Figures 1(c) and 1(d)). Furthermore, qRT-PCR (Figures 1(e) and 1(f)) and western blotting (Figures 1(g)–1(i)) analyses revealed that the mRNA and protein expression levels of the cardiac hypertrophic markers Nppa and Nppb in the TAC+PCI34051 (3, 10, or 30 mg/kg bodyweight/day) groups were downregulated when compared with those in the untreated TAC group.

Next, the effect of PCI34051 on cardiac function was evaluated using echocardiography. At week 6 post-TAC, the lumen of the left ventricle was enlarged, the left ventricle wall was thickened, and the cardiac function was significantly reduced (Supplementary Figure 1). Left ventricular internal dimension end-systolic (LVIDs) and left ventricular internal dimension end-diastolic (LVIDd) were not affected in the sham groups after 8 weeks (6 weeks of TAC+2 weeks of PCI34051 at a dose of 10 mg/kg/day). However, the administration of PCI34051 dose dependently mitigated TAC-induced enhanced LVIDs and LVIDd (Figures 1(j) –1(l)). The effect of PCI34051 at a dose of 30 mg/kg bodyweight/day was higher than that of PCI34051 at doses of 3 and 10 mg/kg bodyweight/day. Fractional shortening (FS) and ejection fraction (EF) were significantly decreased in the TAC groups. However, PCI34051 dose dependently increased FS and EF (Figures 1(m) and 1(n)).

### 3.2. PCI34051 Regulates RAAS Genes in TAC Mice

**Figure 2:**
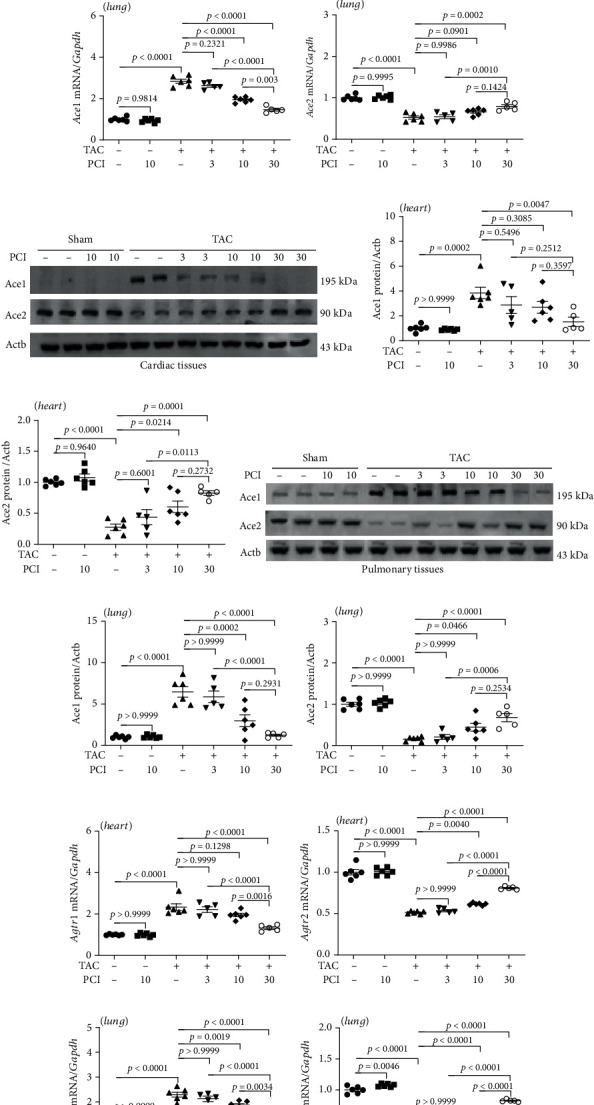
PCI34051 regulates renin-angiotensin system-related genes in the cardiac and pulmonary tissues of transverse aortic constriction (TAC) mice. (a–d) Cardiac and pulmonary mRNA levels of *Ace1* and *Ace2* in the sham+vehicle, sham+PCI34051 (10 mg/kg bodyweight/day), TAC+vehicle, and TAC+PCI34051 (3, 10, or 30 mg/kg bodyweight/day) groups (*n* = 5–6) were examined using quantitative real-time polymerase chain reaction (qRT-PCR). (e) Representative western blot images of Ace1 and Ace2 in cardiac tissues. (f, g) Quantification of Ace1 and Ace2 levels (*n* = 5–6). (h) Representative western blot images of Ace1 and Ace2 in the pulmonary tissues. (i, j) Quantification of Ace1 and Ace2 levels (*n* = 5–6). (k–n) Cardiac and pulmonary mRNA levels of *Agtr1* and *Agtr2* were determined using qRT-PCR and normalized to those of *Gapdh* (*n* = 5–6).

Heart failure is characterized by neurohormonal activation [35, 36]. In this study, we investigated the effects of PCI34051 on the RAAS by evaluating the mRNA expression levels of *Ace1*, *Ace2*, *Agtr1*, and *Agtr2* in cardiac and pulmonary tissues. PCI34051 dose dependently mitigated the TAC-induced upregulation of *Ace1* mRNA levels (Figure 2(a)) and downregulation of *Ace2* mRNA levels in cardiac tissues (Figure 2(b)). Similar *Ace1* and *Ace2* expression patterns were observed in the pulmonary tissues (Figures 2(c) and 2(d)). The expression levels of Ace1 were downregulated in cardiac tissues in the sham group. However, TAC markedly upregulated the levels of Ace1, which was markedly mitigated upon PCI34051 treatment. PCI34051 mitigated the TAC-induced downregulation of Ace2 levels in cardiac tissues (Figures 2(e)–2(g)). Similar Ace1 and Ace2 expression patterns were observed in pulmonary tissues (Figures 2(h)–2(j)). qRT-PCR analysis revealed that PCI34051 mitigated the TAC-induced upregulation of *Agtr1* mRNA levels in cardiac and pulmonary tissues. In contrast, PCI34051 mitigated the TAC-induced downregulation of *Agtr2* mRNA levels (Figures 2(k)–2(n)).

### 3.3. PCI34051 Downregulates the Expression of Hdac8 and Inflammatory Markers in TAC Mice

**Figure 3:**
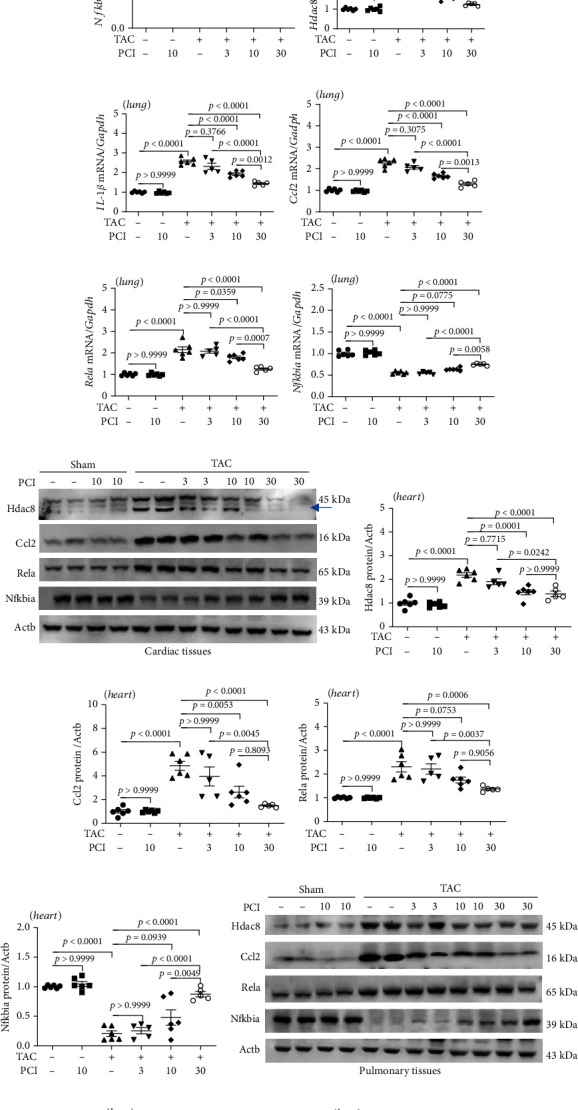
PCI34051 downregulates the expression of Hdac8 and inflammation-related genes in transverse aortic constriction (TAC) mice. (a–j) Cardiac and pulmonary mRNA levels of *Hdac8*, *Il1b*, *Ccl2*, *Rela*, and *Nfkbia* in the sham+vehicle, sham+PCI34051 (10 mg/kg bodyweight/day), TAC+vehicle, and TAC+PCI34051 (3, 10, or 30 mg/kg bodyweight/day) groups were determined using quantitative real-time polymerase chain reaction. (k) Cardiac expression levels of Hdac8, Ccl2, Rela, and Nfkbia; representative western blot images are shown. Actb was used as a loading control. (l–o) Quantification of Hdac8, Ccl2, Rela, and Nfkbia levels (*n* = 5–6 per group). (p) Pulmonary expression levels of Hdac8, Ccl2, Rela, and Nfkbia; representative western blot images. Actb was used as a loading control. (q–t) Quantification of Hdac8, Ccl2, Rela, and Nfkbia levels (*n* = 5–6 per group).

Previous studies have reported that HDAC8 is involved in the induction of cardiac hypertrophy [28, 29]. We hypothesized that heart failure could also be alleviated through HDAC8 inhibition. To verify this hypothesis, mRNA levels of class I HDACs (*Hdac1*, *Hdac*2, *Hdac*3, and *Hdac*8) were determined in TAC mice. TAC did not affect the cardiac and pulmonary levels of *Hdac1*, *Hdac2*, and *Hdac3* (Supplementary Figure 2). However, TAC significantly upregulated the cardiac *Hdac8* mRNA levels. PCI34051 dose dependently mitigated the TAC-induced upregulation of cardiac *Hdac8* mRNA levels (Figure 3(a)). The upregulated levels of inflammatory biomarkers are reported to contribute to heart failure [37]. Therefore, the effect of PCI34051 on inflammatory marker expression was examined. PCI34051 dose dependently mitigated the TAC-induced upregulation of cardiac *Il1b*, *Ccl2*, and *Rela* mRNA levels (Figures 3(b)–3(d)). Additionally, PCI34051 mitigated the TAC-induced downregulation of cardiac *Nfkbia* mRNA levels (Figure 3(e)). The effects of TAC and PCI34051 on the expression patterns of *Hdac8* and inflammation-related genes in the cardiac tissues were similar to those in the pulmonary tissues (Figures 3(f)–3(j)). Western blotting analysis revealed that PCI34051 dose dependently mitigated the TAC-induced upregulation of Hdac8, Ccl2, and Rela in cardiac and pulmonary tissues, which was consistent with the qRT-PCR analysis results (Figures 3(k)–3(t)). PCI34051 mitigated the TAC-induced downregulation of Nfkbia (Figures 3(o) and 3(t)).

### 3.4. PCI34051 Suppresses Cardiac Fibrosis in TAC Mice

**Figure 4:**
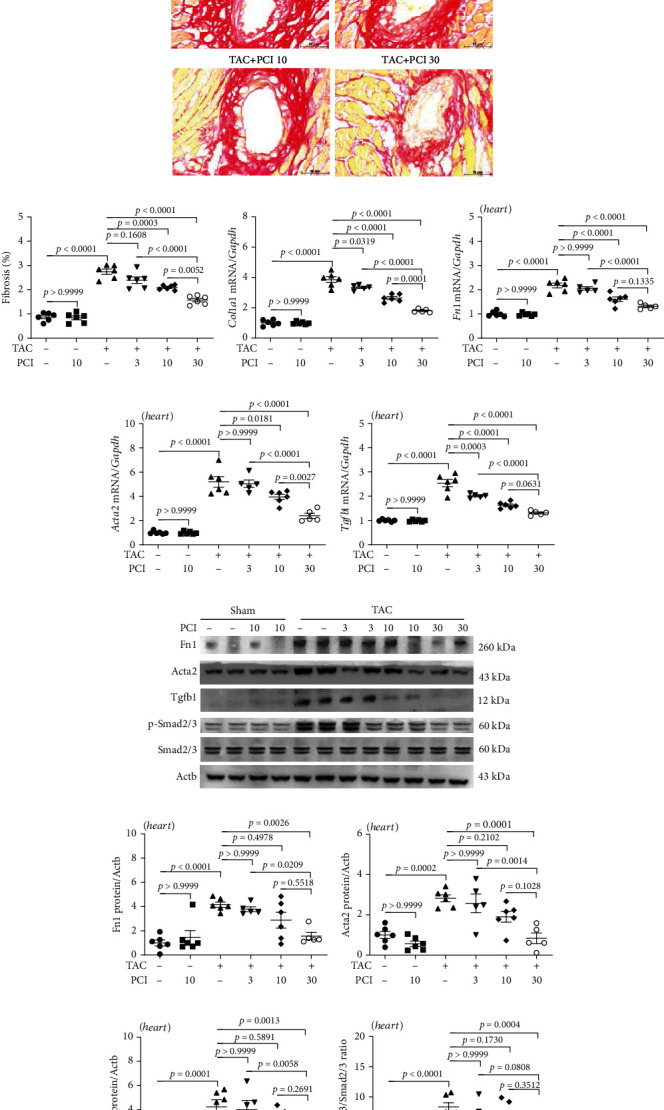
PCI34051 alleviates cardiac fibrosis in transverse aortic constriction (TAC) mice through the TGF-*β*1-Smad2/3 pathway. (a, b) Picrosirius red staining of mouse cardiac tissues; representative images and quantification are shown. Scale bar = 50 *μ*m. The expression levels of the fibrosis marker genes *Col1a1* (c), *Fn1* (d), *Acta2* (e), and *Tgfb1* (f) were determined using quantitative real-time polymerase chain reaction (*n* = 5–6 per group). (g) The cardiac expression levels of Fn1, Acta2, Tgfb1, p-Smad2/3, and Smad2/3 were analyzed using western blotting. Actb was used as a loading control. Representative blots are shown. (h–k) Quantification of Fn1, Acta2, Tgfb1, and p-Smad2/3 to Smad2/3 (*n* = 5–6 per group).

Previous studies have reported that interstitial cardiac fibrosis leads to the development of heart failure [38]. The therapeutic effect of PCI34051 on cardiac fibrosis was examined using Picrosirius red staining, qRT-PCR, and western blotting analyses. As shown in Figures 4(a)nad 4(b), TAC promoted collagen deposition in the perivascular and interstitial regions of the heart. PCI34051 dose dependently decreased TAC-induced collagen accumulation. The cardiac expression levels of the fibrosis-related genes *Col1a1*, *Fn1*, *Acta2*, and *Tgfb1* in the TAC group were upregulated compared with those in the sham group. Treatment with PCI34051 significantly downregulated the expression of these genes (Figures 4(c)–4(f)). The results of western blotting analysis were consistent with those of qRT-PCR analysis (Figures 4(g)–4(j)). TGF-*β*1/Smad2/3 signaling is reported to induce fibrotic gene expression [39]. Hence, the levels of phosphorylated Smad2/3 were examined. Treatment with PCI34051 mitigated the TAC-induced upregulation of cardiac p-Smad2/3 levels (Figures 4(g) and 4(k)).

### 3.5. PCI34051 Alleviates Pulmonary Congestion and Fibrosis in TAC Mice

**Figure 5:**
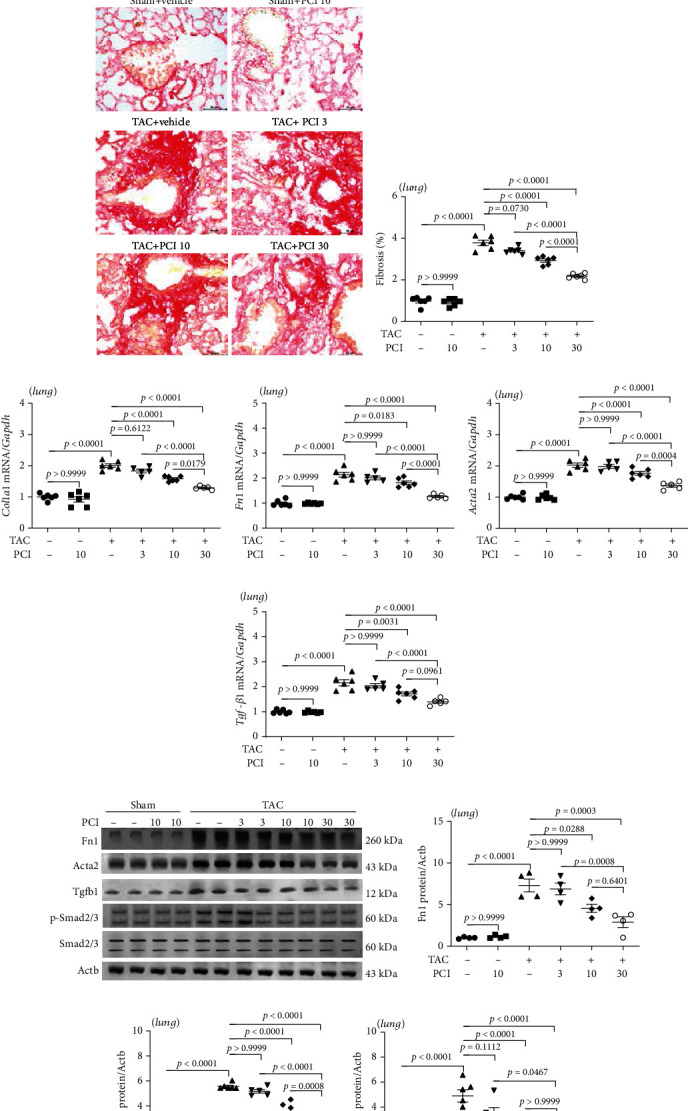
PCI34051 alleviates pulmonary congestion and fibrosis in transverse aortic constriction (TAC) mice. (a) The ratio of lung weight-to-bodyweight (LW/BW) in the sham+vehicle, sham+PCI34051 (10 mg/kg bodyweight/day), TAC+vehicle, and TAC+PCI34051 (3, 10, or 30 mg/kg bodyweight/day) groups (*n* = 5–6 per group) was determined. Representative images of mouse lungs (B). (b) Representative images of pulmonary tissues stained with hematoxylin and eosin. Scale bar = 50 *μ*m. (c) Representative images of pulmonary tissues stained with Picrosirius red. Scale bar = 50 *μ*m. (d) Pulmonary fibrosis was quantified using ImageJ software. (e–h) The mRNA levels of *Col1a1*, *Fn1*, *Acta2*, and *Tgfb1* were evaluated using quantitative real-time polymerase chain reaction (*n* = 5–6 per group). (i–m) Representative western blots and quantification of Fn1, Acta2, Tgfb1, p-Smad2/3, and Smad2/3 levels in the pulmonary tissues (*n* = 5–6 per group).

Heart failure promotes pathological lung remodeling [40]. The effect of PCI34051 on TAC-induced lung remodeling was examined using histological, qRT-PCR, and western blotting analyses. The LW/BW ratio in the TAC group was higher than that in the sham group. PCI34051 significantly and dose dependently mitigated the TAC-induced enhanced LW/BW ratio (Figure 5(a)). H&E staining of pulmonary tissues revealed that the alveolar spaces were not significantly different between the vehicle-treated sham and PCI34051-treated sham groups. PCI34051 mitigated the TAC-induced decreased alveolar spaces (Figure 5(b)). Next, pulmonary fibrosis was evaluated using Picrosirius red staining. Treatment with PCI34051 dose dependently mitigated TAC-induced pulmonary fibrosis (Figurse 5(c) and 5(d)). To further characterize pulmonary fibrosis, the expression levels of fibrosis-related markers were examined using qRT-PCR and western blotting analyses. PCI34051 significantly mitigated the TAC-induced upregulation of *Col1a1*, *Fn1*, *Acta2*, and *Tgfb1* mRNA levels (Figures 5(e)–5(h)). The pulmonary levels of Fn1, Acta2, Tgfb1, and phosphorylated Smad2/3 in the TAC group were upregulated compared with those in the sham group. However, PCI34051 mitigated the TAC-induced upregulation of Fn1, Acta2, Tgfb1, and phosphorylated Smad2/3 in the pulmonary tissues (Figures 5(i)–5(m)).

### 3.6. Knockdown of *Hdac8* or *Ace1* Suppresses TGF-*β*1-Mediated Fibrosis in Primary Rat Cardiac Fibroblasts

**Figure 6:**
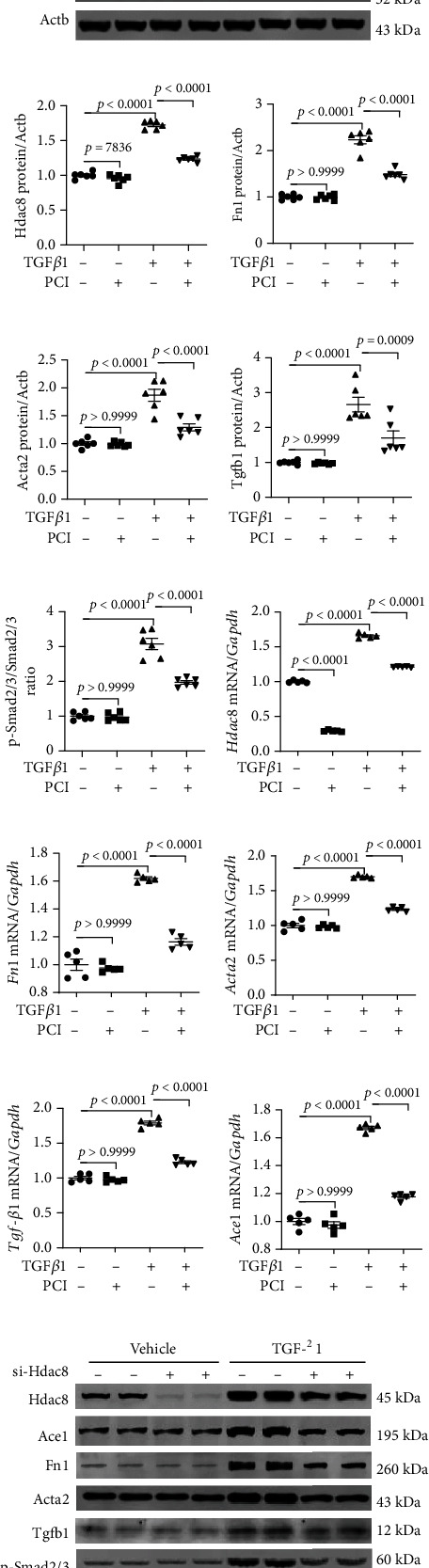
PCI34051 or *Hdac8* knockdown mitigates TGF-*β*1-induced fibrosis in rat cardiac fibroblasts. (a–e) Rat cardiac fibroblasts were pre-treated with TGF–*β*1 (10 ng/mL) for 1 h and cultured in the presence of vehicle or PCI34051 (10 *μ*M) for 8 h. mRNA levels of *Hdac8*, *Col1a1*, *Fn1*, *Acta2*, and *Tgfb1* were evaluated using quantitative real-time polymerase chain reaction (qRT-PCR) (*n* = 4–6 per group). (f–k) Representative immunoblots and quantification of Hdac8, Fn1, Acta2, Tgfb1, p-Smad2/3, and Smad2/3 levels in rat cardiac fibroblasts (*n* = 6 per group). Actb was used as a loading control. (l–p) Rat cardiac fibroblasts were transfected with control or short-interfering RNA against *Hdac8* and treated with TGF-*β*1 (10 ng/mL) for 9 h. The mRNA levels of *Hdac8*, *Fn1*, *Acta2*, *Tgfb1*, and *Ace1* were determined using qRT-PCR (*n* = 5 per group). (q–w) Representative immunoblots and quantification of Hdac8, Ace1, Fn1, Acta2, Tgfb1, p-Smad2/3, and Smad2/3 levels in rat cardiac fibroblasts (*n* = 4 per group). Actb was used as a loading control.

Heart failure is often accompanied by fibrosis, a process characterized by excess production of extracellular matrix in activated fibroblasts [41, 42]. To examine the role of HDAC8 in the regulation of fibrosis in vitro, primary rat cardiac fibroblasts were incubated with PCI34051 in the presence or absence of TGF-*β*1. PCI34051 significantly mitigated the TGF-*β*1-induced upregulation of *Hdac8* mRNA levels (Figure 6(a)). Additionally, PCI34051 mitigated the TGF-*β*1-induced upregulation of the fibrosis-related genes *Col1a1*, *Fn1*, and *Acta2* (Figures 6(b)–6(d)). TGF-*β*1 treatment upregulated the expression of *Tgfb1* (Figure 6(e)). The results of western blotting analysis were consistent with those of qRT-PCR analysis (Figure 6(f)–6(j)). Furthermore, PCI34051 mitigated the TGF-*β*1-induced upregulation of phosphorylated Smad2/3 levels (Figures 6(f) and 6(k)).

To determine whether TGF-*β*1-induced fibrosis was dependent on HDAC8, we transfected H9c2 cells with si-Hdac8. Transfection with si-Hdac8 markedly downregulated the endogenous *Hdac8* mRNA levels (Figure 6(l)) and mitigated the TGF-*β*1-induced upregulation of *Fn1*, *Acta2*, *Tgfb1* (Figures 6(m)–6(o)), and *Ace1* mRNA levels (Figure 6(p)). The results of the western blotting analysis were consistent with those of qRT-PCR analysis (Figures 6(q)–6(v)). Furthermore, transfection with si-Hdac8 mitigated the TGF-*β*1-induced upregulation of phosphorylated Smad2/3 levels (Figures 6(q) and 6(w)).

Next, the role of ACE1 in fibrosis was investigated by downregulating Ace1 expression using siRNAs. Transfection with si-Ace1 markedly downregulated the endogenous *Ace1* mRNA levels, whereas treatment with TGF-*β*1 upregulated the *Ace1* mRNA levels in rat cardiac fibroblasts (Supplementary Figure 3A). Additionally, transfection with si-Ace1 mitigated the TGF-*β*1-induced upregulation of *Hdac8*, *Fn1*, *Acta2*, and *Tgfb1* mRNA levels (Supplementary Figure 3B–E). The results of western blotting analysis were consistent with those of qRT-PCR analysis (Supplementary Figure 3F–K). Furthermore, transfection with si-Ace1 significantly downregulated the levels of phosphorylated Smad2/3 in TGF-*β*1-stimulated fibroblasts (Supplementary Figure 3F and L).

### 3.7. Cardiac Inflammation Is Regulated through the Hdac8-Ace1-Rela-Nfkbia Axis

**Figure 7:**
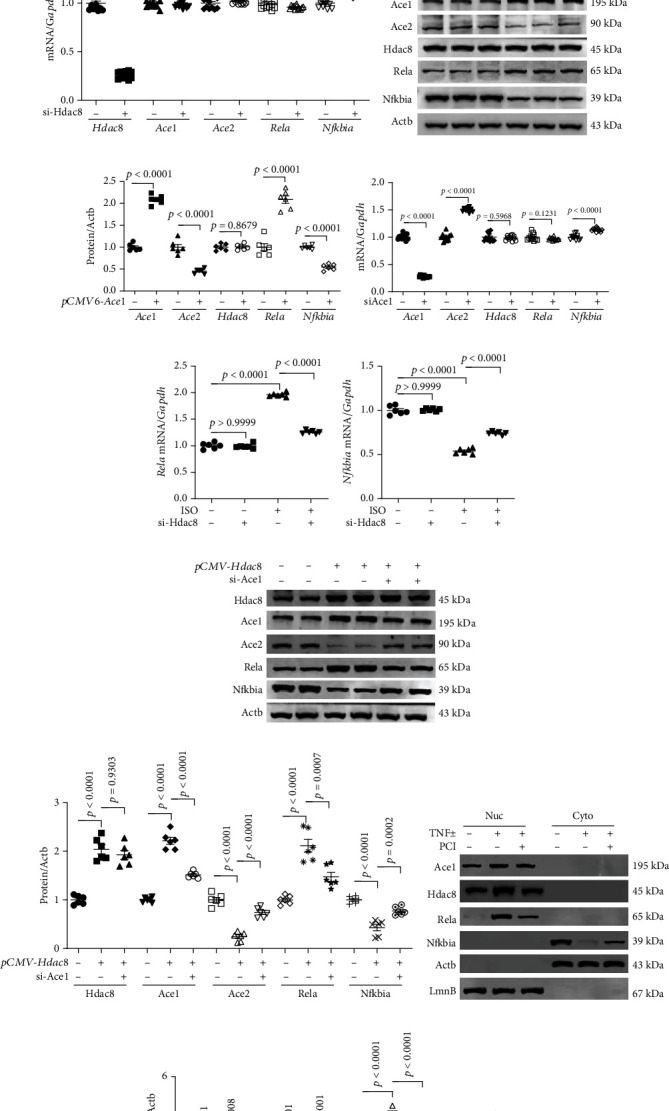
PCI34051 and *Hdac8* or *Ace1* knockdown regulate Rela and Nfkbia expression in cardiomyocytes. (a) mRNA levels of *Hdac8*, *Ace1*, *Ace2*, *Rela*, and *Nfkbia* in H9c2 cells transfected with *pCMV* vector or *pCMV-Hdac8* were evaluated using quantitative real-time polymerase chain reaction (qRT-PCR) (*n* = 6 per group). (b) mRNA levels of *Hdac8*, *Ace1*, *Ace2*, *Rela*, and *Nfkbia* in H9c2 cells transfected with control short-interfering RNA (siRNA) or siRNA against *Hdac8* (si-Hdac8) were evaluated using qRT-PCR (*n* = 12 per group). (c, d) Representative immunoblots and quantification of Ace1, Ace2, Hdac8, Rela, and Nfkbia levels in *pCMV6-Ace1*-transfected H9c2 cells (*n* = 6 per group). Actb was used as a loading control. (e) mRNA levels of *Ace1*, *Ace2*, *Hdac8*, *Rela*, and *Nfkbia* in H9c2 cells transfected with control siRNA or si-Ace1 were evaluated using qRT-PCR (*n* = 12 per group). (f, g) The mRNA levels of *Rela* and *Nfkbia* in H9c2 cells transfected with control siRNA or si-Hdac8 and treated with isoproterenol (10 *μ*M) for 9 h were measured using qRT-PCR (*n* = 6 per group). (h, i) The expression levels of Hdac8, Ace1, Ace2, Rela, and Nfkbia in H9c2 cells transfected with *pCMV* or *pCMV-Hdac8* and control siRNA or si-Ace1; representative immunoblots and quantification of protein levels are shown (*n* = 6 per group). Actb was used as a loading control. (j, k) H9c2 cells were incubated with TNF*α* (50 ng/mL) for 1 h and cultured in the presence of vehicle or PCI34051 (10 *μ*M) for 5 h. The nuclear and cytoplasmic fractions were obtained as described in Materials and Methods. Representative western blots and quantification of protein levels (*n* = 4 per group). Actb and LmnB were used as loading controls for the cytoplasmic and nuclear fractions, respectively.

To elucidate the regulatory mechanisms of PCI34051, the role of Hdac8 and Ace1 in inflammation was examined. H9c2 cardiomyocytes were transfected with *pCMV* or *pCMV-Hdac8* constructs. Hdac8 overexpression upregulated *Hdac8* and *Ace1* mRNA levels and downregulated *Ace2* mRNA levels. The NF-*κ*B signaling pathway is reported to play a key role in various inflammatory diseases [43]. Hdac8 overexpression significantly upregulated *Rela* mRNA levels and downregulated *Nfkbia* mRNA levels (Figure 7(a)). Next, the expression of Hdac8 was knocked down using siRNA. Transfection with si-Hdac8 markedly decreased endogenous *Hdac8* mRNA levels but did not affect *Ace1* and *Ace2* mRNA levels. Additionally, transfection with si-Hdac8 downregulated *Rela* mRNA levels and upregulated *Nfkbia* mRNA levels (Figure 7(b)). To investigate the role of Ace1, the cells were transfected with *pCMV6* or *pCMV6-Ace1* constructs. Ace1 overexpression upregulated endogenous Ace1 levels and downregulated Ace2 levels. However, Ace1 overexpression did not affect Hdac8 levels. Additionally, Ace1 overexpression significantly upregulated Rela levels and downregulated Nfkbia levels (Figures 7(c) and 7(d)). Meanwhile, *Ace1* knockdown downregulated endogenous *Ace1* mRNA levels and upregulated *Ace2* mRNA levels. However, *Ace1* knockdown did not affect *Hdac8* mRNA levels but significantly upregulated *Nfkbia* mRNA levels (Figure 7(e)). To evaluate the effect of *Hdac8* knockdown on isoproterenol-induced inflammation, H9c2 cardiomyocytes were transfected with control siRNA or si-Hdac8 and treated with isoproterenol. Transfection with si-Hdac8 did not affect *Ace1* and *Ace2* mRNA levels. Additionally, si-Hdac8 mitigated the isoproterenol-induced upregulation of *Ace1* mRNA levels and downregulation of *Ace2* mRNA levels (Supplementary Figure 4A–B). Furthermore, si-Hdac8 mitigated the isoproterenol-induced upregulation of *Rela* mRNA levels and downregulation of *Nfkbia* mRNA levels (Figures 7(f) and 7(g)). Next, the role of ACE1 in HDAC8-mediated inflammation response was examined. H9c2 cells were transfected with *pCMV-Hdac8* construct and si-Ace1. Transfection with si-Ace1 did not affect Hdac8 levels, which suggested that *Ace1* is a downstream gene of Hdac8. Hdac8 overexpression mitigated si-Ace1-induced upregulation of Ace2. Furthermore, si-Ace1 mitigated Hdac8 overexpression-induced Rela upregulation and Nfkbia downregulation (Figures 7(h) and 7(i)). To analyze the association between HDAC8 and inflammation, H9c2 cells were incubated with PCI34051 in the presence or absence of TNF*α*. The localization of NF-*κ*B (nuclear vs. cytoplasmic) was evaluated. Treatment with TNF-*α* did not affect the localization of Ace1 and Hdac8, which were localized in the nuclear fraction. PCI34051 mitigated the TNF-*α*-induced upregulation of nuclear Rela levels and downregulation of cytosolic Nfkbia levels (Figures 7(j) and 7(k)). These findings indicate that PCI34051 exerts cardioprotective effects through the downregulation of the NF-*κ*B signaling pathway.

## 4. Discussion

**Figure 8:**
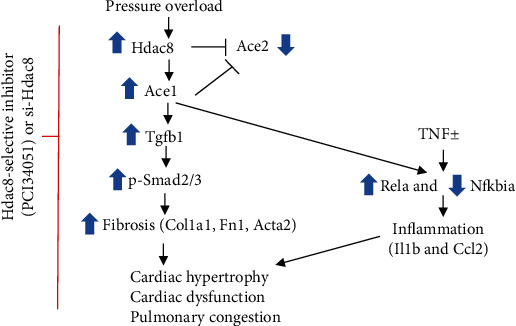
Suppression of HDAC8 attenuates fibrosis, inflammation, cardiac dysfunction, and pulmonary congestion in heart failure. Schematic diagram depicting the Hdac8 selective inhibitor- (PCI34051-) mediated or Hdac8 short-interfering RNA-mediated inhibition of transverse aortic constriction- (TAC-) induced heart failure. TAC-mediated pressure overload upregulates the expression of Hdac8 and Ace1 and downregulates the expression of Ace2 in the heart and lungs. In cardiomyocytes, overexpression of Hdac8 upregulates the expression of Ace1 and downregulates the expression of Ace2. PCI34051 treatment or *Hdac8* knockdown mitigates TGF-*β*1-induced upregulation of p-Smad2/3, Col1a1, Fn1, and Acta2 in vitro. PCI34051 regulates the TNF*α*-mediated or Ace1 overexpression-mediated expression of Rela and Nfkbia. PCI34051 alleviates cardiac hypertrophy and dysfunction, inflammation, fibrosis, and pulmonary congestion in heart failure.

In this study, we found that the selective HDAC8 inhibitor (PCI34051) alleviated pathological heart and lung conditions, mitigated cardiac dysfunction, and suppressed inflammation and fibrosis in TAC mice through the Hdac8-Ace1 axis (Figure 8).

The cardiac and pulmonary expression levels of Hdac8 but not those of other class I HDACs (HDAC1, HDAC2, and HDAC3) were upregulated in TAC mice. This suggested a distinct role of HDAC8 in the regulation of heart failure. The expression levels of Ace1 and Agtr1 were upregulated, whereas those of Ace2 and Agtr2 were downregulated in the cardiac and pulmonary tissues of TAC mice. Treatment with PCI34051 mitigated the TAC-induced changes in the expression levels of these genes, which suggested that HDAC8 promoted the activation of RAAS during heart failure. Hdac8 overexpression upregulated Ace1 and Agtr1 levels and downregulated Ace2 and Agtr2 levels in cardiomyocytes (Figure 7(a) and Supplementary Figure 5). Previous studies have reported that RAAS is activated in patients with heart failure [44]. However, the direct roles of RAAS components in the pathogenesis of heart failure are unclear. This study demonstrated that overexpression of Ace1 and Hdac8 upregulated the expression of inflammatory markers in cardiomyocytes, which was mitigated upon *Ace1* knockdown. ACE1 and ACE2 are reported to play an important role in inflammation [45, 46]. Therefore, the expression levels of Ace1 and Ace2 were examined in the TAC-induced mouse model. Overexpression of Hdac8 or Ace1 downregulated Ace2 levels, which suggested that Hdac8 is a novel negative regulator of Ace2 expression.

Recently, we reported that PCI34051 attenuates catecholamine-induced cardiac hypertrophy [28]. In this study, PCI34051 dose dependently alleviated cardiac hypertrophy and downregulated the expression of hypertrophy-specific genes (*Nppa* and *Nppb*) in the cardiac tissues of TAC mice. The TAC mouse model is characterized by decreased systolic function and development of heart failure with reduced EF [47]. PCI34051 regulated cardiac remodeling (increased the size of the left ventricular lumen) and consequently improved cardiac function. Heart failure is usually accompanied by cardiac hypertrophy. The role of HDAC2, which is the best characterized HDAC, in cardiac hypertrophy has been previously reported [20–22, 48, 49]. For example, SK-7041, a highly selective inhibitor of HDAC1 and HDAC2, alleviated cardiac hypertrophy in the rat TAC model [23]. Although HDAC2 and HDAC8 promote cardiac hypertrophy, the underlying mechanisms may vary. The enzymatic activity of HDAC2 was reported to be critical for the development of cardiac hypertrophy [21], whereas upregulation of HDAC8 expression was critical for promoting cardiac hypertrophy. Moreover, cardiac Hdac2 and Hdac8 expression levels are upregulated during cardiac remodeling in renovascular hypertensive rats [50]. One study reported that the expression levels of HDAC1, HDAC2, and HDAC8 were upregulated in patients with idiopathic pulmonary arterial hypertension [51]. Zhang et al. reported that calcium calmodulin kinase II promoted cardiac dysfunction by activating HDAC1 and HDAC3 [52]. However, these results are not consistent with some previous studies, which reported that HDAC3 is involved in postnatal cardiac myocyte proliferation and not cardiac hypertrophy [26]. The findings of this study suggested that sustained upregulation of Hdac8 mRNA and protein levels has a critical role in the transition from cardiac hypertrophy to heart failure.

Next, the expression levels of inflammation-related genes in the cardiac and pulmonary tissues of TAC mice were examined. TAC upregulated Rela levels and downregulated Nfkbia levels. Treatment with PCI34051 mitigated Rela upregulation and Nfkbia downregulation in vivo and in vitro. Additionally, PCI34051 mitigated the TNF*α*-induced nuclear localization of Rela, Ace1, and Hdac8 and downregulation of Nfkbia in the cytosol of cardiomyocytes. These findings indicate that PCI34051 exerts inhibitory effects on inflammatory signaling. Interestingly, the expression levels of *Rela* and *Nfkbia* were not affected in TGF-*β*1-stimulated cardiac fibroblasts (Supplementary Figure 6). Previously, we had demonstrated that PCI34051 downregulated the expression of inflammatory markers and adhesion molecules in hypertension [27]. WK2-16, an HDAC8 inhibitor, is reported to alleviate neuroinflammation both in vivo and in vitro [53]. CCL2, an inflammatory cytokine, plays an important role in the pathogenesis of heart failure [54]. In this study, PCI34051 mitigated the heart failure-induced upregulation of Ccl2.

Furthermore, the results of Picrosirius red staining revealed that PCI34051 dose dependently mitigated TAC-induced fibrosis in the mouse cardiac and pulmonary tissues. This indicated that HDAC8 is a potential novel therapeutic target for fibrosis. TGF-*β*1, a fibrogenic growth factor, upregulated the expression of *Hdac8* and fibrosis-related genes. The expression levels of Tgfb1 and phosphorylated Smad2/3 were upregulated in TAC mice and TGF-*β*1-treated cardiac fibroblasts. The antifibrotic effect of PCI34051 or *Hdac8* knockdown was potentially mediated through inhibition of the TGF-*β*1-Smad2/3 pathway. Interestingly, TGF-*β*1-induced fibrosis was dependent on Ace1. The expression levels of Fn1 and Acta2 were downregulated in TGF-*β*1-treated *Ace1* knockdown cardiac fibroblasts (Supplementary Figure 3). Moreover, the mRNA and protein levels of Ace1 were markedly upregulated in cardiac and pulmonary tissues of TAC mice. These observations suggest that the activation of RAAS contributed to pressure overload-induced fibrosis. The findings of this study are consistent with those in previous reports, which reported that ACE1 was upregulated and ACE2 was downregulated in bleomycin-induced lung fibrosis [55].

Cardiomyocytes were treated with actinomycin D, a transcription inhibitor, to elucidate the mechanisms underlying Hdac8 overexpression-mediated upregulation of Ace1 and Rela, downregulation of Ace2 and Nfkbia, and regulation of inflammation and fibrosis. Actinomycin D mitigated Hdac8 overexpression-induced upregulation of Ace1 and Rela. This indicated that Hdac8 upregulated the expression levels of Ace1 and Rela at the transcript level (Supplementary Figure 7).

## 5. Conclusions

This study demonstrated that the HDAC8 selective inhibitor PCI34051 alleviated cardiac hypertrophy, pulmonary congestion, inflammation, and fibrosis, which resulted in improved cardiac functions in the pressure overload-induced heart failure mouse model. Among class I HDACs, only Hdac8 was upregulated in the cardiac and pulmonary tissues of the heart failure mouse model. Additionally, this study demonstrated that Hdac8 and Ace1 are involved in the proinflammatory Rela pathway and the fibrosis-related TGF-*β*1-Smad2/3 pathway. Thus, HDAC8 is a potential novel therapeutic target for heart failure.

## 6. Limitations of the Study

Here, we showed that the selective HDAC8 inhibitor PCI34051 alleviated heart failure. However, the inhibitory effects of PCI34051 on other types of HDACs cannot be ruled out. Therefore, future studies must confirm the therapeutic effects of PCI34051 on heart failure using the *Hdac8* knockout mouse model. Additionally, we used the cardiomyocyte cell line to determine the effects of Hdac8 or Ace1 overexpression on the expression of inflammation-related genes. HDAC8 regulates cardiac hypertrophy through the AKT/GSK3*β* pathway, which was not investigated in this study. Furthermore, this study used the H9c2 cell line to investigate the mechanisms of TGF-*β*1-induced fibrosis. In contrast to primary cardiomyocytes, the H9c2 cells are not beating cells. However, the hypertrophic responses of H9c2 cells are similar to those of primary cardiomyocytes.

## Data Availability

The data used to support the findings of this study are included within the supplementary information file.

## Supplementary Materials

Supplementary Materials Supplementary Figure 1: echocardiography parameters in mice at week 6 posttransverse aortic constriction (TAC). (A) Echocardiography parameters in mice belonging to the sham and TAC groups at week 6 post-TAC. Quantification of (A) left ventricular internal diameter end-systole (LVIDs, mm), (B) left ventricular internal diameter end-diastole (LVIDd, mm), (C) interventricular septum (IVSd, mm), (D) left ventricular posterior wall thickness (LVPWd, mm), (E) fractional shortening (FS, %), and (F) ejection fraction (EF, %), (*n* = 5–6). Data are presented as mean ± standard error and analyzed using one-way analysis of variance, followed by Bonferroni post hoc test. Supplementary Figure 2: cardiac and pulmonary mRNA expression levels of class I histone deacetylases (HDACs) in transverse aortic constriction (TAC) mice. The cardiac (A–C) and pulmonary (D–F) mRNA levels of *Hdac1*, *Hdac2*, and *Hdac3* in the sham, TAC, and TAC+PCI34051 (3, 10, or 30 mg/kg bodyweight/day) groups were examined using quantitative real-time polymerase chain reaction. The expression levels of target genes were normalized to those of *Gapdh.* Data are presented as mean ± standard error and analyzed using one-way analysis of variance, followed by Bonferroni post hoc test. Supplementary Figure 3: Ace1 knockdown downregulates the expression of fibrosis-related genes in primary rat cardiac fibroblasts. (A–E) Rat cardiac fibroblasts transfected with control or short-interfering RNAs against *Ace1* (si-Ace1) were incubated with TGF-*β*1. The mRNA levels of *Ace1*, *Hdac8*, *Fn1*, *Acta2*, and *Tgfb1* were determined using quantitative real-time polymerase chain reaction. (F–L) Representative blots and quantification of Ace1, Hdac8, Fn1, Acta2, Tgfb1, p-Smad2/3, and Smad2/3 levels in the cardiac tissues. Actb was used as a loading control. Data are presented as mean ± standard error and analyzed using one-way analysis of variance, followed by Bonferroni post hoc test. Supplementary Figure 4: Hdac8 knockdown regulates Ace1 and Ace2 mRNA levels in isoproterenol-treated H9c2 cells. (A, B) H9c2 cells transfected with control or short-interfering RNAs against *Hdac8* (si-Hdac8) were incubated with isoproterenol. The mRNA levels of *Ace1* and *Ace2* were determined using quantitative real-time polymerase chain reaction. The expression levels of target genes were normalized to those of *Gapdh*. Data are presented as mean ± standard error and analyzed using one-way analysis of variance, followed by Bonferroni *post hoc* test. Supplementary Figure 5: Hdac8 overexpression upregulates Agtr1 and downregulates Agtr2 in H9c2 cells. (A, B) H9c2 cells were transfected with an empty vector or a *pCMV-Hdac8* construct. The mRNA levels of *Agtr1* and *Agtr2* were determined using quantitative real-time polymerase chain reaction. Supplementary Figure 6: PCI34051 treatment or Hdac8 and Ace1 knockdown did not affect the expression of Rela and Nfkbia in rat cardiac fibroblasts. (A–B) Rat cardiac fibroblasts were treated with vehicle or PCI34051 in the presence or absence of TGF-*β*1. The mRNA levels of *Rela* and *Nfkbia* were determined using quantitative real-time polymerase chain reaction (qRT-PCR). (C–D) Rat cardiac fibroblasts transfected with control or short-interfering RNAs against *Hdac8* (si-Hdac8) were treated with TGF-*β*1. The mRNA levels of *Rela* and *Nfkbia* were determined using qRT-PCR. (E–F) Rat cardiac fibroblasts transfected with control or si-Ace1 were treated with TGF-*β*1. The mRNA levels of *Rela* and *Nfkbia* were determined using qRT-PCR. Data are presented as the mean ± standard error and analyzed using one-way analysis of variance, followed by Bonferroni *post hoc* test. Supplementary Figure 7: Hdac8 upregulated the expression of Ace1 and Rela at the transcript level. (A−E) H9c2 cells transfected with vector or *pCMV-Hdac8* were treated with actinomycin D (2.5 *μ*g/mL) for 6 h. The mRNA levels of *Hdac8*, *Ace1*, *Ace2*, *Rela*, and *Nfkbia* were determined using quantitative real-time polymerase chain reaction. Data are presented as mean ± standard error and analyzed using one-way analysis of variance, followed by Bonferroni *post hoc* test. Supplementary Table 1: primers for the RT-PCR.
