# Supplementary figures and images for: Hdac8 Inhibitor Alleviates Transverse Aortic Constriction-Induced Heart Failure in Mice by Downregulating Ace1
Source: Oxid Med Cell Longev. 2022 Jan 27;2022:6227330. doi: 10.1155/2022/6227330 (PMC8813277; doi:10.1155/2022/6227330)

## Slide 1
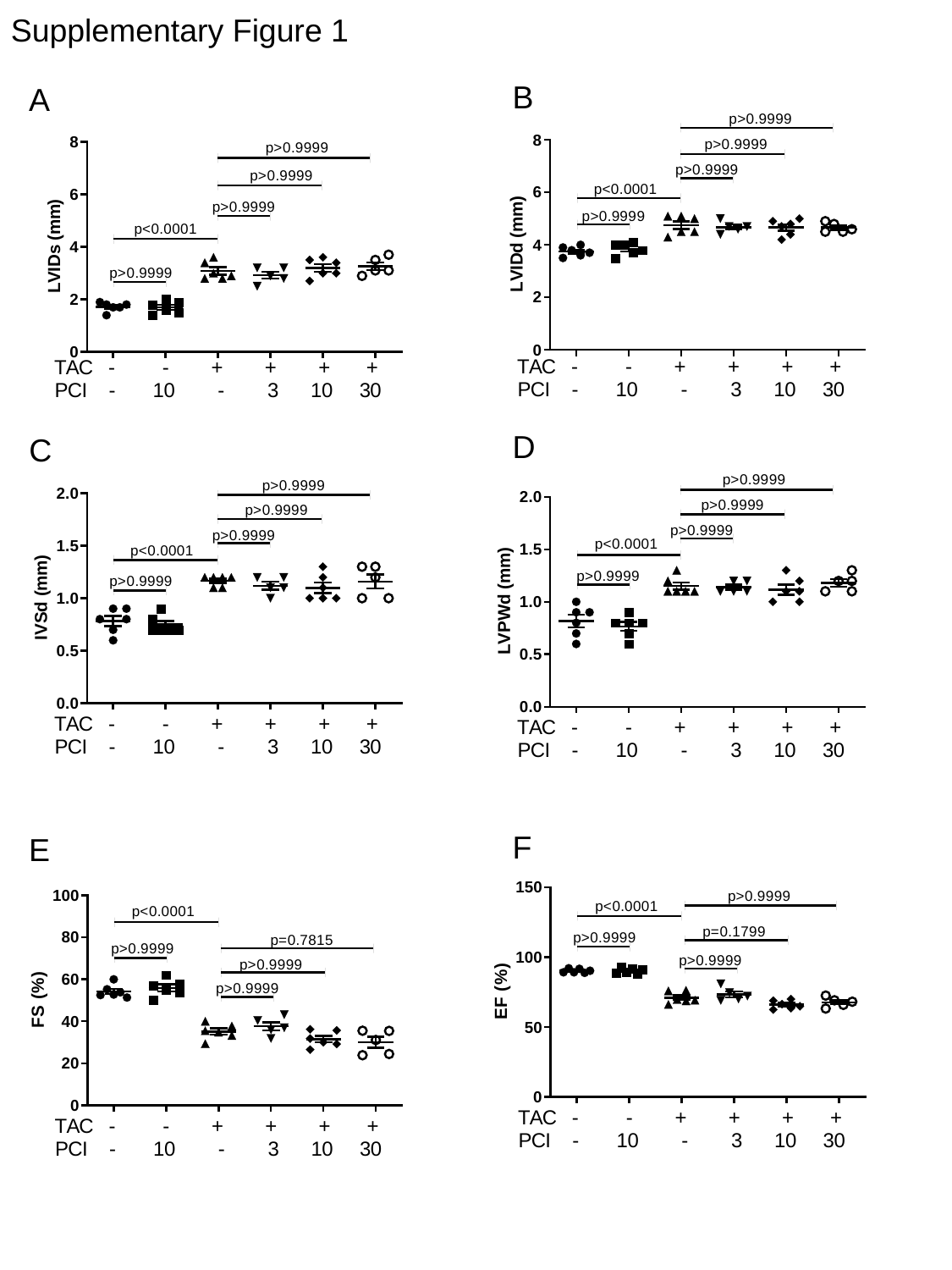

Supplementary Figure 1
B
A
D
C
F
E

Supplement: Supplementary Materials — Supplementary Figure 1: echocardiography parameters in mice at week 6 posttransverse aortic constriction (TAC). (A) Echocardiography parameters in mice belonging to the sham and TAC groups at week 6 post-TAC. Quantification of (A) left ventricular internal diameter end-systole (LVIDs, mm), (B) left ventricular internal diameter end-diastole (LVIDd, mm), (C) interventricular septum (IVSd, mm), (D) left ventricular posterior wall thickness (LVPWd, mm), (E) fractional shortening (FS, %), and (F) ejection fraction (EF, %), (n = 5–6). Data are presented as mean ± standard error and analyzed using one-way analysis of variance, followed by Bonferroni post hoc test. Supplementary Figure 2: cardiac and pulmonary mRNA expression levels of class I histone deacetylases (HDACs) in transverse aortic constriction (TAC) mice. The cardiac (A–C) and pulmonary (D–F) mRNA levels of Hdac1, Hdac2, and Hdac3 in the sham, TAC, and TAC+PCI34051 (3, 10, or 30 mg/kg bodyweight/day) groups were examined using quantitative real-time polymerase chain reaction. The expression levels of target genes were normalized to those of Gapdh. Data are presented as mean ± standard error and analyzed using one-way analysis of variance, followed by Bonferroni post hoc test. Supplementary Figure 3: Ace1 knockdown downregulates the expression of fibrosis-related genes in primary rat cardiac fibroblasts. (A–E) Rat cardiac fibroblasts transfected with control or short-interfering RNAs against Ace1 (si-Ace1) were incubated with TGF-β1. The mRNA levels of Ace1, Hdac8, Fn1, Acta2, and Tgfb1 were determined using quantitative real-time polymerase chain reaction. (F–L) Representative blots and quantification of Ace1, Hdac8, Fn1, Acta2, Tgfb1, p-Smad2/3, and Smad2/3 levels in the cardiac tissues. Actb was used as a loading control. Data are presented as mean ± standard error and analyzed using one-way analysis of variance, followed by Bonferroni post hoc test. Supplementary Figure 4: Hdac8 knockdown regulates Ace1 and [file 6227330.f1.zip › Revision_supplementary Figure1.pptx]

## Slide 1
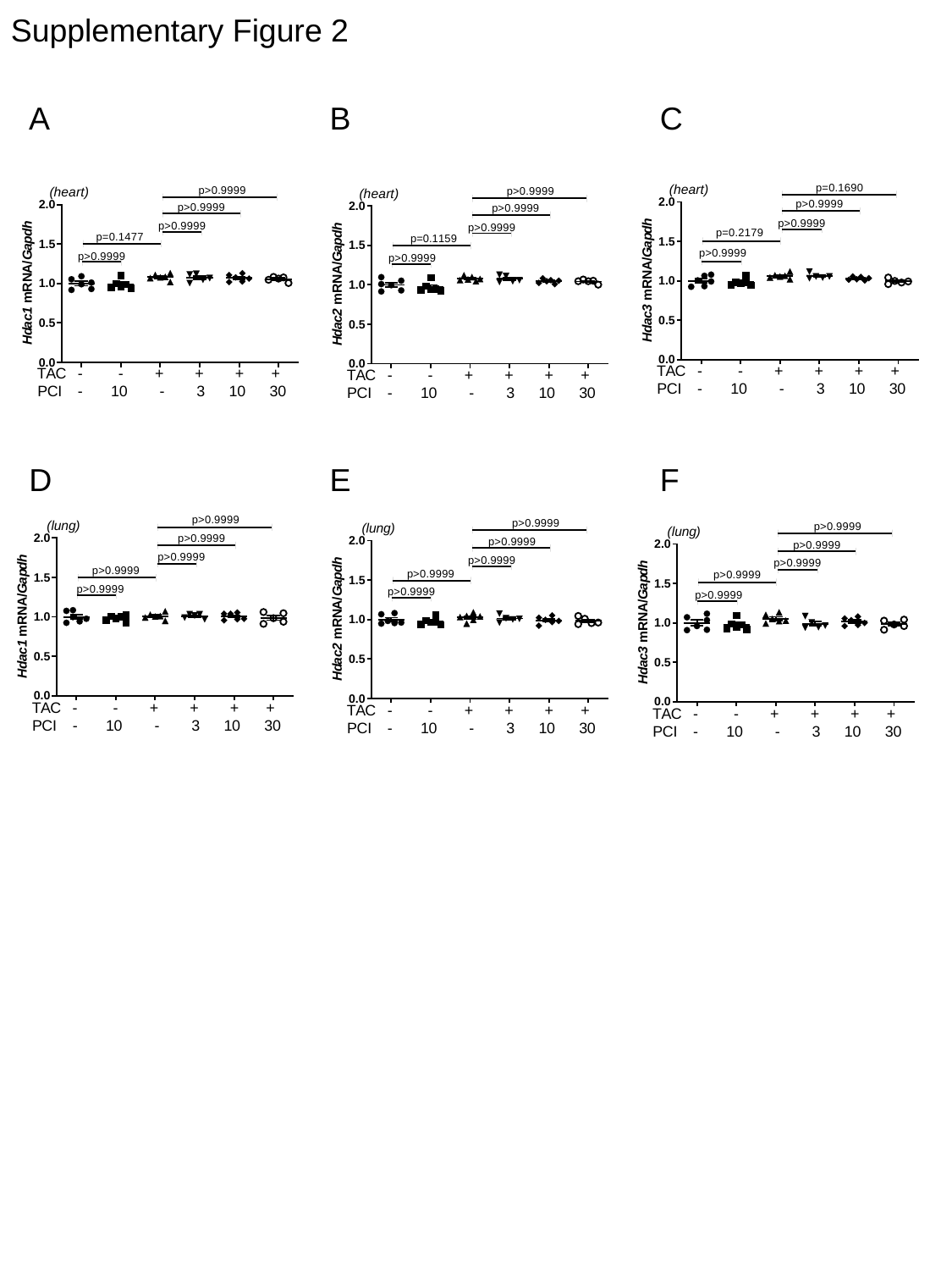

Supplementary Figure 2
A
B
C
D
E
F

Supplement: Supplementary Materials — Supplementary Figure 1: echocardiography parameters in mice at week 6 posttransverse aortic constriction (TAC). (A) Echocardiography parameters in mice belonging to the sham and TAC groups at week 6 post-TAC. Quantification of (A) left ventricular internal diameter end-systole (LVIDs, mm), (B) left ventricular internal diameter end-diastole (LVIDd, mm), (C) interventricular septum (IVSd, mm), (D) left ventricular posterior wall thickness (LVPWd, mm), (E) fractional shortening (FS, %), and (F) ejection fraction (EF, %), (n = 5–6). Data are presented as mean ± standard error and analyzed using one-way analysis of variance, followed by Bonferroni post hoc test. Supplementary Figure 2: cardiac and pulmonary mRNA expression levels of class I histone deacetylases (HDACs) in transverse aortic constriction (TAC) mice. The cardiac (A–C) and pulmonary (D–F) mRNA levels of Hdac1, Hdac2, and Hdac3 in the sham, TAC, and TAC+PCI34051 (3, 10, or 30 mg/kg bodyweight/day) groups were examined using quantitative real-time polymerase chain reaction. The expression levels of target genes were normalized to those of Gapdh. Data are presented as mean ± standard error and analyzed using one-way analysis of variance, followed by Bonferroni post hoc test. Supplementary Figure 3: Ace1 knockdown downregulates the expression of fibrosis-related genes in primary rat cardiac fibroblasts. (A–E) Rat cardiac fibroblasts transfected with control or short-interfering RNAs against Ace1 (si-Ace1) were incubated with TGF-β1. The mRNA levels of Ace1, Hdac8, Fn1, Acta2, and Tgfb1 were determined using quantitative real-time polymerase chain reaction. (F–L) Representative blots and quantification of Ace1, Hdac8, Fn1, Acta2, Tgfb1, p-Smad2/3, and Smad2/3 levels in the cardiac tissues. Actb was used as a loading control. Data are presented as mean ± standard error and analyzed using one-way analysis of variance, followed by Bonferroni post hoc test. Supplementary Figure 4: Hdac8 knockdown regulates Ace1 and [file 6227330.f1.zip › Revision_supplementary Figure2.pptx]

## Slide 1
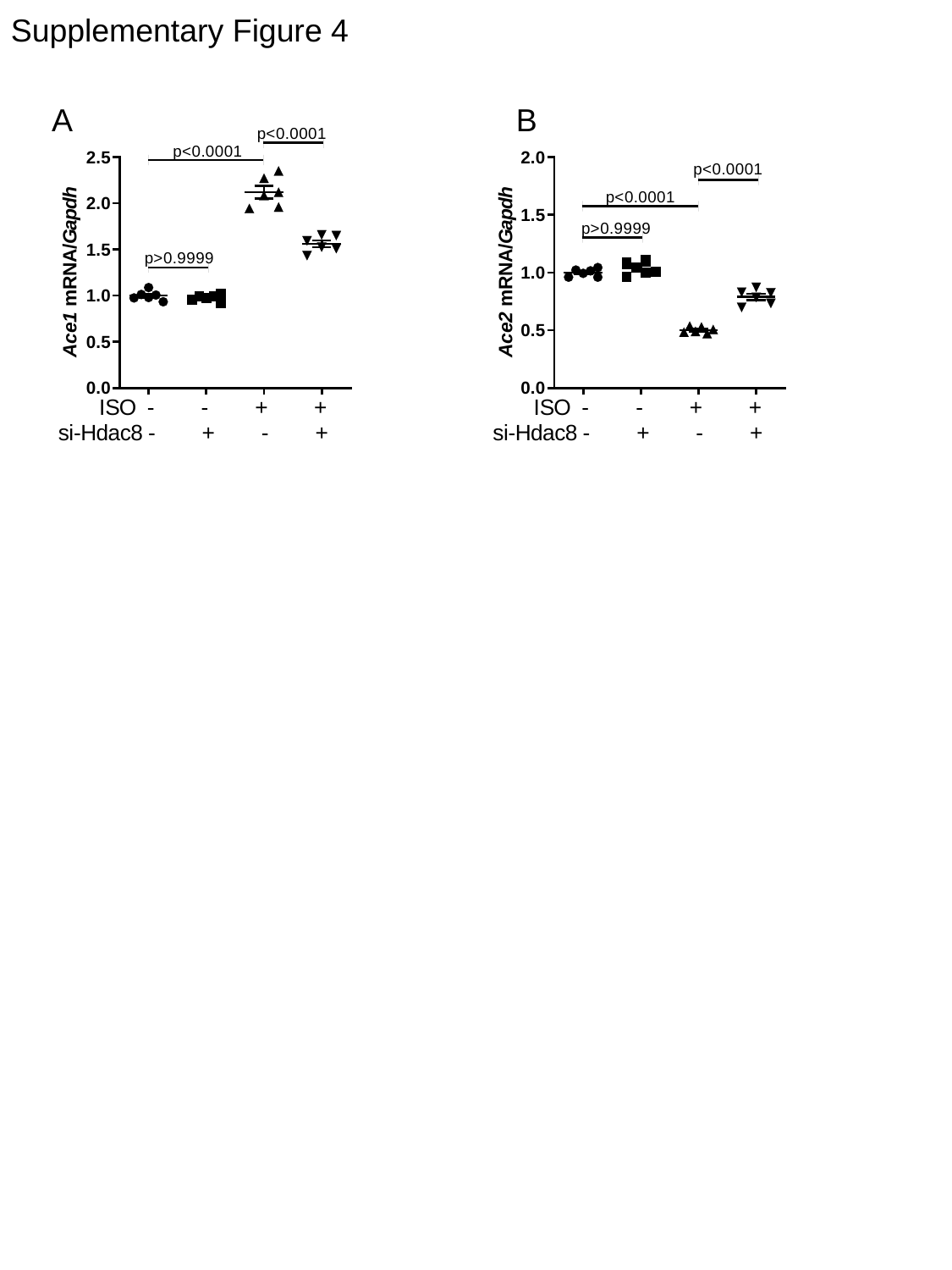

Supplementary Figure 4
A
B

Supplement: Supplementary Materials — Supplementary Figure 1: echocardiography parameters in mice at week 6 posttransverse aortic constriction (TAC). (A) Echocardiography parameters in mice belonging to the sham and TAC groups at week 6 post-TAC. Quantification of (A) left ventricular internal diameter end-systole (LVIDs, mm), (B) left ventricular internal diameter end-diastole (LVIDd, mm), (C) interventricular septum (IVSd, mm), (D) left ventricular posterior wall thickness (LVPWd, mm), (E) fractional shortening (FS, %), and (F) ejection fraction (EF, %), (n = 5–6). Data are presented as mean ± standard error and analyzed using one-way analysis of variance, followed by Bonferroni post hoc test. Supplementary Figure 2: cardiac and pulmonary mRNA expression levels of class I histone deacetylases (HDACs) in transverse aortic constriction (TAC) mice. The cardiac (A–C) and pulmonary (D–F) mRNA levels of Hdac1, Hdac2, and Hdac3 in the sham, TAC, and TAC+PCI34051 (3, 10, or 30 mg/kg bodyweight/day) groups were examined using quantitative real-time polymerase chain reaction. The expression levels of target genes were normalized to those of Gapdh. Data are presented as mean ± standard error and analyzed using one-way analysis of variance, followed by Bonferroni post hoc test. Supplementary Figure 3: Ace1 knockdown downregulates the expression of fibrosis-related genes in primary rat cardiac fibroblasts. (A–E) Rat cardiac fibroblasts transfected with control or short-interfering RNAs against Ace1 (si-Ace1) were incubated with TGF-β1. The mRNA levels of Ace1, Hdac8, Fn1, Acta2, and Tgfb1 were determined using quantitative real-time polymerase chain reaction. (F–L) Representative blots and quantification of Ace1, Hdac8, Fn1, Acta2, Tgfb1, p-Smad2/3, and Smad2/3 levels in the cardiac tissues. Actb was used as a loading control. Data are presented as mean ± standard error and analyzed using one-way analysis of variance, followed by Bonferroni post hoc test. Supplementary Figure 4: Hdac8 knockdown regulates Ace1 and [file 6227330.f1.zip › Revision_supplementary Figure4.pptx]

## Slide 1
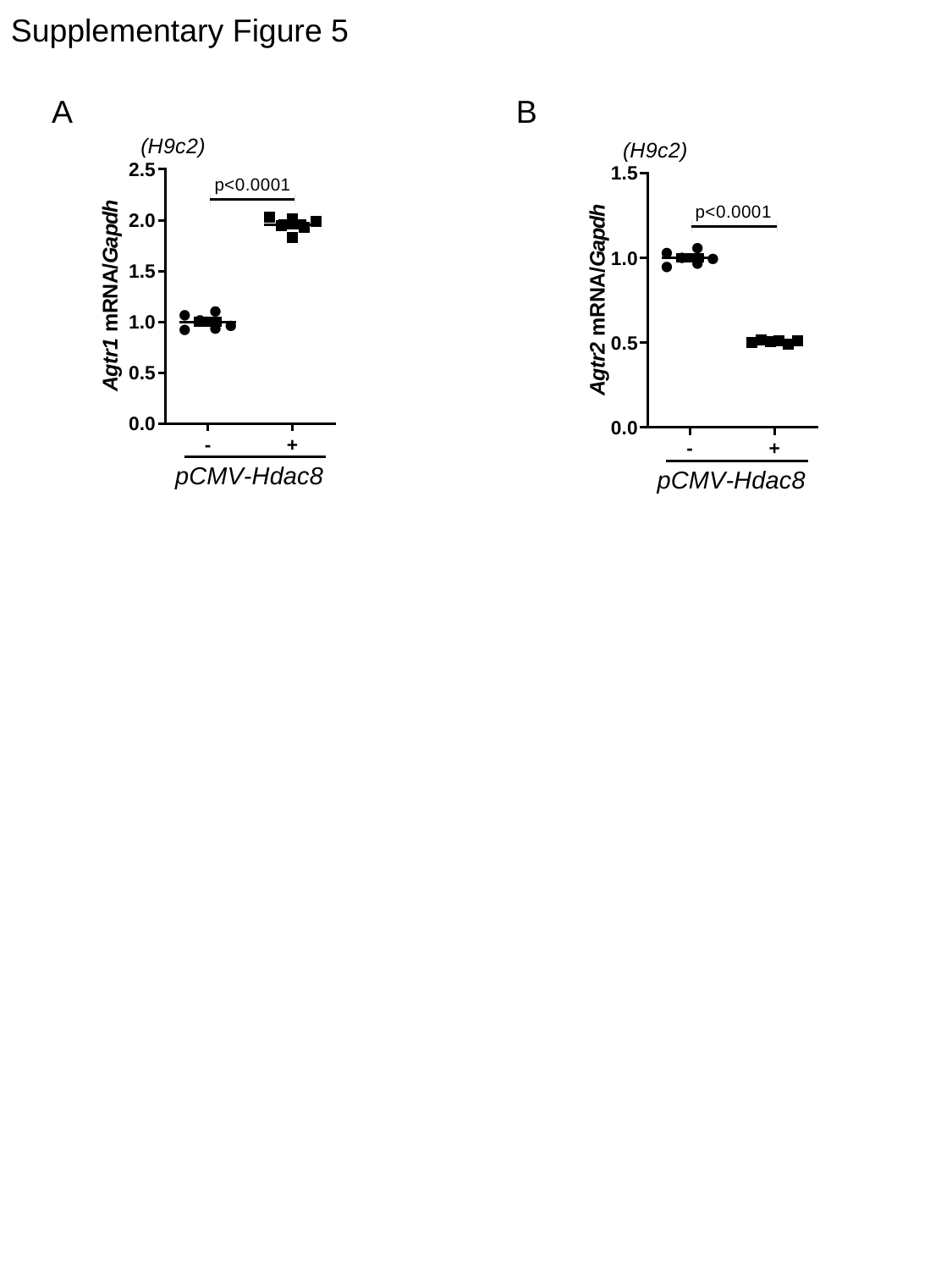

Supplementary Figure 5
A
B

Supplement: Supplementary Materials — Supplementary Figure 1: echocardiography parameters in mice at week 6 posttransverse aortic constriction (TAC). (A) Echocardiography parameters in mice belonging to the sham and TAC groups at week 6 post-TAC. Quantification of (A) left ventricular internal diameter end-systole (LVIDs, mm), (B) left ventricular internal diameter end-diastole (LVIDd, mm), (C) interventricular septum (IVSd, mm), (D) left ventricular posterior wall thickness (LVPWd, mm), (E) fractional shortening (FS, %), and (F) ejection fraction (EF, %), (n = 5–6). Data are presented as mean ± standard error and analyzed using one-way analysis of variance, followed by Bonferroni post hoc test. Supplementary Figure 2: cardiac and pulmonary mRNA expression levels of class I histone deacetylases (HDACs) in transverse aortic constriction (TAC) mice. The cardiac (A–C) and pulmonary (D–F) mRNA levels of Hdac1, Hdac2, and Hdac3 in the sham, TAC, and TAC+PCI34051 (3, 10, or 30 mg/kg bodyweight/day) groups were examined using quantitative real-time polymerase chain reaction. The expression levels of target genes were normalized to those of Gapdh. Data are presented as mean ± standard error and analyzed using one-way analysis of variance, followed by Bonferroni post hoc test. Supplementary Figure 3: Ace1 knockdown downregulates the expression of fibrosis-related genes in primary rat cardiac fibroblasts. (A–E) Rat cardiac fibroblasts transfected with control or short-interfering RNAs against Ace1 (si-Ace1) were incubated with TGF-β1. The mRNA levels of Ace1, Hdac8, Fn1, Acta2, and Tgfb1 were determined using quantitative real-time polymerase chain reaction. (F–L) Representative blots and quantification of Ace1, Hdac8, Fn1, Acta2, Tgfb1, p-Smad2/3, and Smad2/3 levels in the cardiac tissues. Actb was used as a loading control. Data are presented as mean ± standard error and analyzed using one-way analysis of variance, followed by Bonferroni post hoc test. Supplementary Figure 4: Hdac8 knockdown regulates Ace1 and [file 6227330.f1.zip › Revision_supplementary Figure5.pptx]

## Slide 1
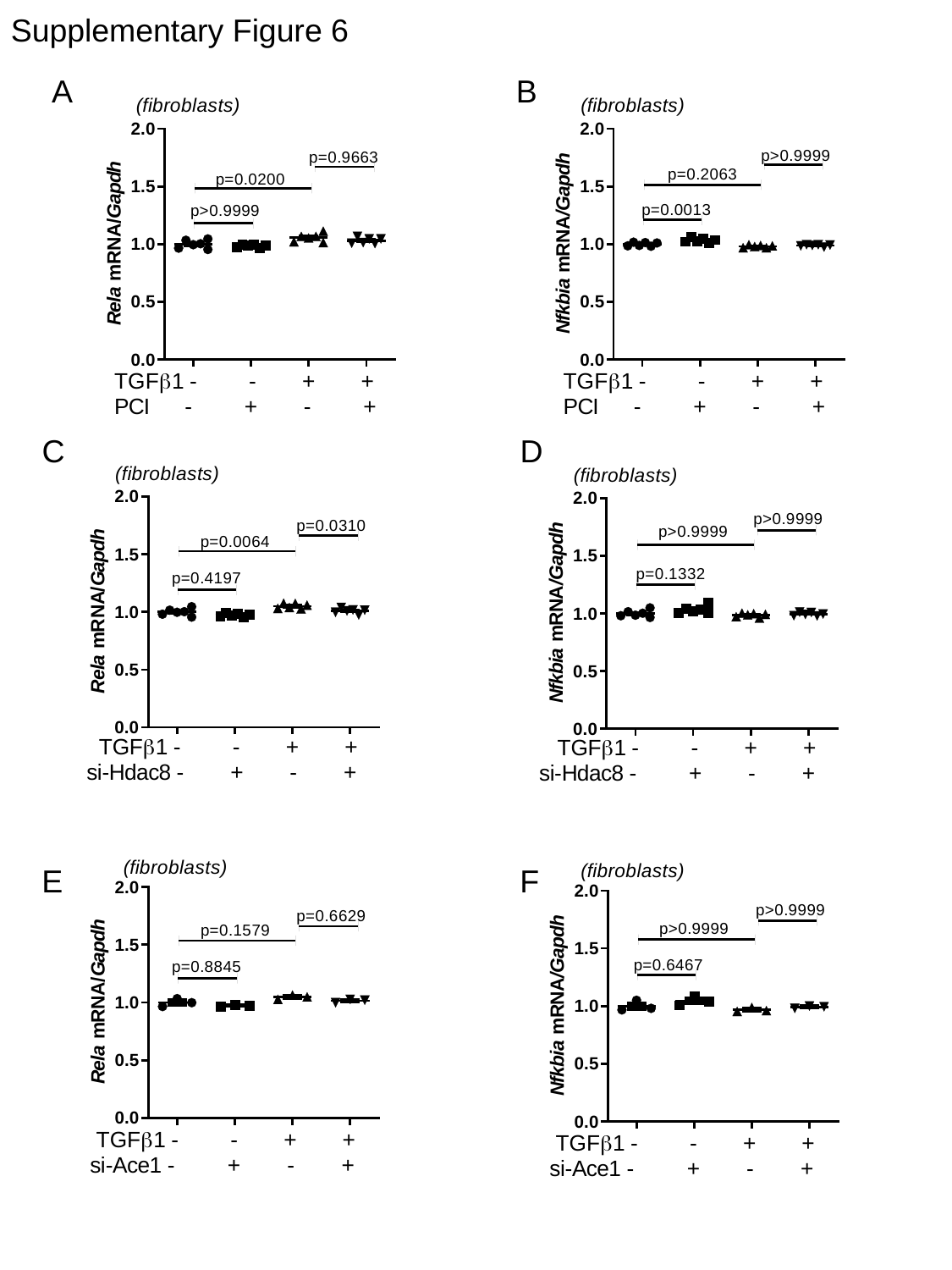

Supplementary Figure 6
A
B
C
D
E
F

Supplement: Supplementary Materials — Supplementary Figure 1: echocardiography parameters in mice at week 6 posttransverse aortic constriction (TAC). (A) Echocardiography parameters in mice belonging to the sham and TAC groups at week 6 post-TAC. Quantification of (A) left ventricular internal diameter end-systole (LVIDs, mm), (B) left ventricular internal diameter end-diastole (LVIDd, mm), (C) interventricular septum (IVSd, mm), (D) left ventricular posterior wall thickness (LVPWd, mm), (E) fractional shortening (FS, %), and (F) ejection fraction (EF, %), (n = 5–6). Data are presented as mean ± standard error and analyzed using one-way analysis of variance, followed by Bonferroni post hoc test. Supplementary Figure 2: cardiac and pulmonary mRNA expression levels of class I histone deacetylases (HDACs) in transverse aortic constriction (TAC) mice. The cardiac (A–C) and pulmonary (D–F) mRNA levels of Hdac1, Hdac2, and Hdac3 in the sham, TAC, and TAC+PCI34051 (3, 10, or 30 mg/kg bodyweight/day) groups were examined using quantitative real-time polymerase chain reaction. The expression levels of target genes were normalized to those of Gapdh. Data are presented as mean ± standard error and analyzed using one-way analysis of variance, followed by Bonferroni post hoc test. Supplementary Figure 3: Ace1 knockdown downregulates the expression of fibrosis-related genes in primary rat cardiac fibroblasts. (A–E) Rat cardiac fibroblasts transfected with control or short-interfering RNAs against Ace1 (si-Ace1) were incubated with TGF-β1. The mRNA levels of Ace1, Hdac8, Fn1, Acta2, and Tgfb1 were determined using quantitative real-time polymerase chain reaction. (F–L) Representative blots and quantification of Ace1, Hdac8, Fn1, Acta2, Tgfb1, p-Smad2/3, and Smad2/3 levels in the cardiac tissues. Actb was used as a loading control. Data are presented as mean ± standard error and analyzed using one-way analysis of variance, followed by Bonferroni post hoc test. Supplementary Figure 4: Hdac8 knockdown regulates Ace1 and [file 6227330.f1.zip › Revision_supplementary Figure6.pptx]

## Slide 1
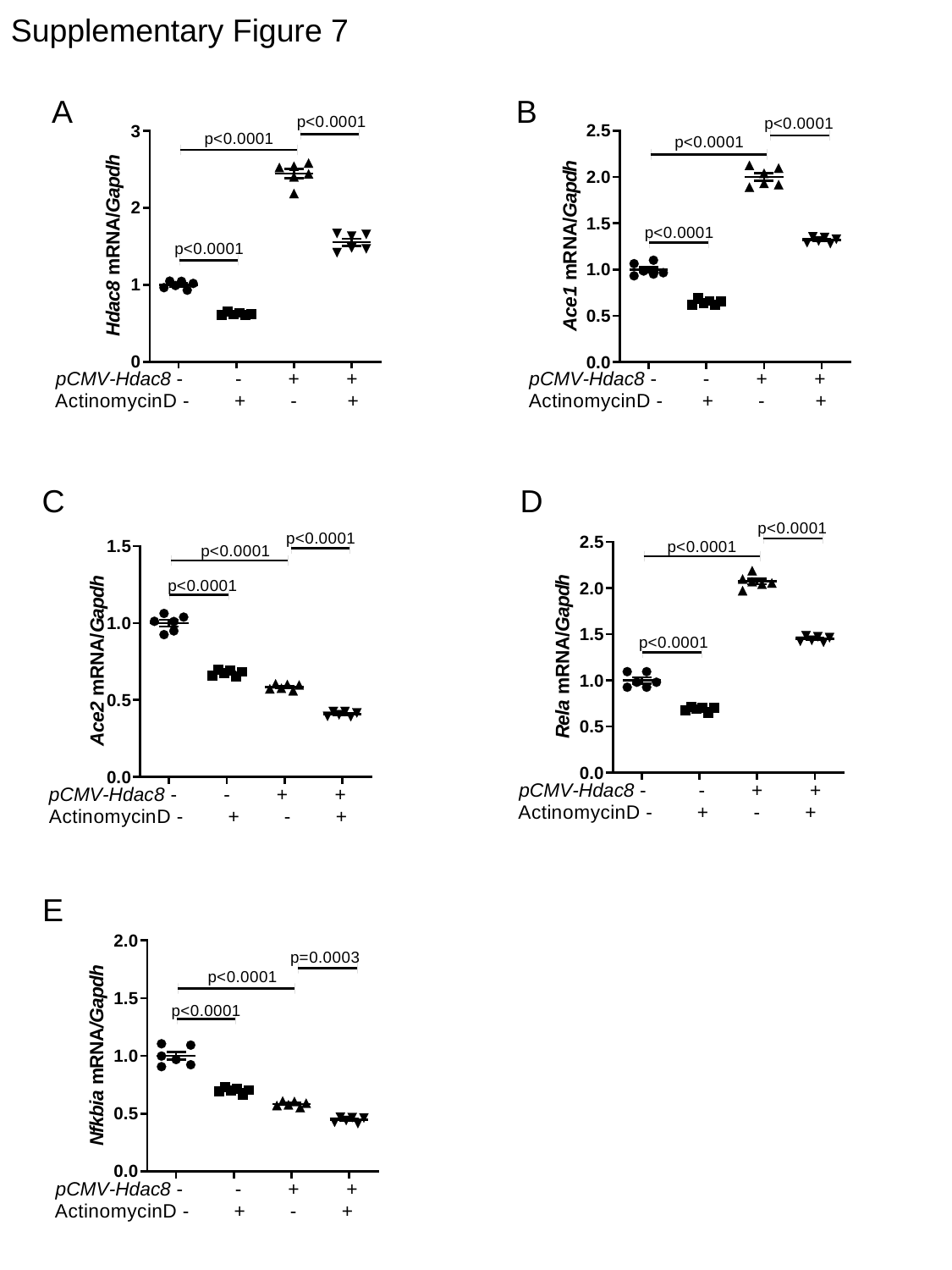

Supplementary Figure 7
A
B
C
D
E

Supplement: Supplementary Materials — Supplementary Figure 1: echocardiography parameters in mice at week 6 posttransverse aortic constriction (TAC). (A) Echocardiography parameters in mice belonging to the sham and TAC groups at week 6 post-TAC. Quantification of (A) left ventricular internal diameter end-systole (LVIDs, mm), (B) left ventricular internal diameter end-diastole (LVIDd, mm), (C) interventricular septum (IVSd, mm), (D) left ventricular posterior wall thickness (LVPWd, mm), (E) fractional shortening (FS, %), and (F) ejection fraction (EF, %), (n = 5–6). Data are presented as mean ± standard error and analyzed using one-way analysis of variance, followed by Bonferroni post hoc test. Supplementary Figure 2: cardiac and pulmonary mRNA expression levels of class I histone deacetylases (HDACs) in transverse aortic constriction (TAC) mice. The cardiac (A–C) and pulmonary (D–F) mRNA levels of Hdac1, Hdac2, and Hdac3 in the sham, TAC, and TAC+PCI34051 (3, 10, or 30 mg/kg bodyweight/day) groups were examined using quantitative real-time polymerase chain reaction. The expression levels of target genes were normalized to those of Gapdh. Data are presented as mean ± standard error and analyzed using one-way analysis of variance, followed by Bonferroni post hoc test. Supplementary Figure 3: Ace1 knockdown downregulates the expression of fibrosis-related genes in primary rat cardiac fibroblasts. (A–E) Rat cardiac fibroblasts transfected with control or short-interfering RNAs against Ace1 (si-Ace1) were incubated with TGF-β1. The mRNA levels of Ace1, Hdac8, Fn1, Acta2, and Tgfb1 were determined using quantitative real-time polymerase chain reaction. (F–L) Representative blots and quantification of Ace1, Hdac8, Fn1, Acta2, Tgfb1, p-Smad2/3, and Smad2/3 levels in the cardiac tissues. Actb was used as a loading control. Data are presented as mean ± standard error and analyzed using one-way analysis of variance, followed by Bonferroni post hoc test. Supplementary Figure 4: Hdac8 knockdown regulates Ace1 and [file 6227330.f1.zip › Revision_supplementary Figure7.pptx]
