# Supplementary material for: Hdac8 Inhibitor Alleviates Transverse Aortic Constriction-Induced Heart Failure in Mice by Downregulating Ace1
Source: Oxid Med Cell Longev. 2022 Jan 27;2022:6227330. doi: 10.1155/2022/6227330 (PMC8813277; doi:10.1155/2022/6227330)
Supplement: Supplementary Materials — Supplementary Figure 1: echocardiography parameters in mice at week 6 posttransverse aortic constriction (TAC). (A) Echocardiography parameters in mice belonging to the sham and TAC groups at week 6 post-TAC. Quantification of (A) left ventricular internal diameter end-systole (LVIDs, mm), (B) left ventricular internal diameter end-diastole (LVIDd, mm), (C) interventricular septum (IVSd, mm), (D) left ventricular posterior wall thickness (LVPWd, mm), (E) fractional shortening (FS, %), and (F) ejection fraction (EF, %), (n = 5–6). Data are presented as mean ± standard error and analyzed using one-way analysis of variance, followed by Bonferroni post hoc test. Supplementary Figure 2: cardiac and pulmonary mRNA expression levels of class I histone deacetylases (HDACs) in transverse aortic constriction (TAC) mice. The cardiac (A–C) and pulmonary (D–F) mRNA levels of Hdac1, Hdac2, and Hdac3 in the sham, TAC, and TAC+PCI34051 (3, 10, or 30 mg/kg bodyweight/day) groups were examined using quantitative real-time polymerase chain reaction. The expression levels of target genes were normalized to those of Gapdh. Data are presented as mean ± standard error and analyzed using one-way analysis of variance, followed by Bonferroni post hoc test. Supplementary Figure 3: Ace1 knockdown downregulates the expression of fibrosis-related genes in primary rat cardiac fibroblasts. (A–E) Rat cardiac fibroblasts transfected with control or short-interfering RNAs against Ace1 (si-Ace1) were incubated with TGF-β1. The mRNA levels of Ace1, Hdac8, Fn1, Acta2, and Tgfb1 were determined using quantitative real-time polymerase chain reaction. (F–L) Representative blots and quantification of Ace1, Hdac8, Fn1, Acta2, Tgfb1, p-Smad2/3, and Smad2/3 levels in the cardiac tissues. Actb was used as a loading control. Data are presented as mean ± standard error and analyzed using one-way analysis of variance, followed by Bonferroni post hoc test. Supplementary Figure 4: Hdac8 knockdown regulates Ace1 and [file 6227330.f1.zip › Supplementary Table 1.doc]

**Supplementary Table 1. Primers for the RT-PCR**

| Gene | Primer sequence (5′ to 3′) |
| --- | --- |
| *Gapdh (mouse)* | F: GCATGGCCTTCCGTGTTCCT  R: CCCTGTTGCTGTAGCCGTATTCAT |
| *Nppa (mouse)* | F: TGGAGGAGAAGATGCCGGTAGAAGAT  R: AGCGAGCAGAGCCCTCAGTTTGCT |
| *Nppb (mouse)* | F: CTGAAGGTGCTGTCCCAGAT  R: GTTCTTTTGTGAGGCCTTGG |
| *Ace1 (mouse)* | F: CAGTGTCTACCCCCAAGCAT  R: TTCCATCAAAGACCCTCCAG |
| *Ace2 (mouse)* | F: CTACAGGCCCTTCAGCAAAG  R: TGCCCAGAGCCTAGAGTTGT |
| *Agtr1 (mouse)* | F: GGAAACAGCTTGGTGGTGAT  R: TGAGACACGTGAGCAGGAAC |
| *Agtr2 (mouse)* | F: CCCTGGCAAGCATCTTATGT  R: CCAGCAGACCACTGAGCATA |
| *Hdac1 (mouse)* | F: TGCTGTGAACTACCCACTGC  R: CACGAACTCCACACACTTGG |
| *Hdac2 (mouse)* | F: GCTGGGGCTGTGAAATTAAA  R: CTTCCTCAACACCATCACCA |
| *Hdac3 (mouse)* | F: AGCTGGACACCCAATGAAAC  R: CTCTTGGTGAAACCCTGCAT |
| *Hdac8 (mouse)* | F: TCCGAAGGCAGTGGTTTTAC  R: GATGACCCCGGTCAAGTATG |
| *Il1b (mouse)* | F: GCCCATCCTCTGTGACTCAT  R: AGGCCACAGGTATTTTGTCG |
| *Ccl2 (mouse)* | F: AGGTCCCTGTCATGCTTCTG  R: TCTGGACCCATTCCTTCTTG |
| *Rela (mouse)* | F: TCAATGGCTACACAGGACCA  R: CTAATGGCTTGCTCCAGGTC |
| *Nfkbia (mouse)* | F: AGGACGAGGAGTACGAGCAA  R: GTCTCCCTTCACCTGACCAA |
| *Col1a1 (mouse)* | F: GAGCGGAGAGTACTGGATCG  R: GCTTCTTTTCCTTGGGGTTC |
| *Fn1 (mouse)* | F: GATGCACCGATTGTCAACAG  R: TGATCAGCATGGACCACTTC |
| *Acta2 (mouse)* | F: ACTGGGACGACATGGAAAAG  R: AGAGGCATAGAGGGACAGCA |
| *Tgfb1 (mouse)* | F: CTCTCCACCTGCAAGACCAT  R: ACGCGGGTGACCTCTTTAG |
| *Gapdh (rat)* | F: AACCCATCACCATCTTCCAGGAGC  R: ATGGACTGTGGTCATGAGCCCTTC |
| *Hdac8 (rat)* | F: CGCTACCCCCGGTTTATATT  R: CTTCTTGGCTGACCTTCTGG |
| *Fn1 (rat)* | F: AGCAAATCGTGCAGCCTCCG  R: CCCCCTTCATGGCAGCGATT |
| *Acta2 (rat)* | F: GACACCAGGGAGTGATGGTT  R: GTTAGCAAGGTCGGATGCTC |
| *Tgfb1 (rat)* | F: TGCTTCAGCTCCACAGAGAA  R: TGGTTGTAGAGGGCAAGGAC |
| *Ace1 (rat)* | F: GTACAGAAGGGCTGGAATGC  R: CGTGCACTGCTTAATCCTGA |
| *Ace2 (rat)* | F: TCCTTCTCAGCCTTGTTGCT  R: TCATCTTTTGGGCATTCTCC |
| *Rela (rat)* | F: ACATGAACTTGTGGGGAAGG  R: CACTGTCACCTGGAAGCAGA |
| *Nfkbia (rat)* | F: TTGGTCAGGTGAAGGGAGAC  R: GGCAAGATGGAGAGGGGTAT |
| *Agtr1 (rat)* | F: ACCAGGTCAAGTGGATTTCG  R: ATCACCACCAAGCTGTTTCC |
| *Agtr2 (rat)* | F: CCTTCTTGGATGCTCTGACC  R: TGGAGCCAAGTAATGGGAAC |
